# Biochemical and Nutritional Characterization of Edible Seaweeds from the Peruvian Coast

**DOI:** 10.3390/plants12091795

**Published:** 2023-04-27

**Authors:** Natalia Arakaki, Leenin Flores Ramos, Alberto Isidoro Oscanoa Huaynate, Anthony Ruíz Soto, María Eliana Ramírez

**Affiliations:** 1 Banco de Germoplasma de Organismos Acuáticos, Área Funcional de Investigaciones en Acuicultura, Instituto del Mar del Perú, Esquina Gamarra y General Valle S/N, Chucuito, Callao 07021, Peru; 2 Laboratorio de Análisis Instrumental, Área Funcional de Investigaciones en Acuicultura, Instituto del Mar del Perú, Esquina Gamarra y General Valle S/N, Chucuito, Callao 07021, Peruaoscanoa@imarpe.gob.pe (A.I.O.H.); aruiz@imarpe.gob.pe (A.R.S.); 3 Museo Nacional de Historia Natural, Área Botánica, Casilla 787, Santiago 8500000, Chile; mramirezcasali@gmail.com

**Keywords:** macroalgae, proximate composition, amino acid, fatty acid, Peru

## Abstract

In Peru, the number of species of edible seaweeds within the genera *Chondracanthus*, *Porphyra* (hereafter *P.*), *Pyropia* (hereafter *Py.*), and *Ulva* has not been fully established, nor is there a significant level of information available related to their chemical and nutritional composition. This study involved the biochemical analysis of species belonging to ten genera of macroalgae, known edible and some of which have the potential to be used as food, including six red (*Callophyllis, Chondracanthus, Mazzaella*, *Porphyra*, *Pyropia*, and *Rhodymenia*), two green (*Ulva* and *Codium*), and two brown (*Eisenia* and *Lessonia*) species collected along the Peruvian coast (6°–17° S). In the evaluation of 37 specimens, differences were found in the proximal composition, amino acid composition, and fatty acid profiles, which were specific to subgroups and supported their taxonomic classification, mainly at the order level. The red algae *Porphyra/Pyropia* (Bangiales) had the highest average percentage of protein (24.10%) and carbohydrates (59.85%) and the lowest percentage of ash (7.95%). Conversely, the brown alga *Eisenia* (Laminariales) had the lowest average percentage of protein, with different values related to the structure: 14.11% at the level of the frond and 9.46% at the level of the stipe. On the other hand, Bryopsidales green algae showed the highest average percentages of lipids (5.38%). The moisture percentages ranged from 4 to 16%, and no relevant significant differences were shown between the orders. The characteristic amino acids in all of the studied groups were glutamic acid, aspartic acid, alanine, and leucine. The highest average of the essential amino acids ratio was obtained for the Gigartinales red algae (48.65%), and the highest values of the essential amino acid index (EAAI) were obtained for the Ulvales, Laminariales, Gigartinales, and Rhodymeniales algae (EAAI > 0.92). The highest average relative percentage of fatty acids was obtained for polyunsaturated fatty acids, followed by saturated fatty acids. The major component of the ω6 fatty acids from red and brown algae was arachidonic acid (C20:4n − 6). The highest level of ω3 fatty acids was observed for the eicosapentaenoic acids (EPA) in red algae. The highest median ω6/ω3 ratio was displayed by the red alga *Callophyllis variegata* (Gigartinales). A detailed knowledge of edible seaweeds, and those considered potentially edible, would help to diversify the diet based on macroalgae in Peru.

## 1. Introduction

In contrast to European and American cultures, edible seaweeds have a long history in the diets of Asian cultures, in which algae are used in food production and various industrial applications due to their thickening and gelling properties [1,2,3]. In Latin America, consuming seaweed is not common, and the biomass harvested is limited to uses in the phycocolloid industry [4]. However, there is a growing interest in the use of edible seaweeds in functional foods and in gastronomy at all levels [5]. Hence, in tropical and subtropical countries, algae with high biomasses are being recognized as having nutritional value and can be cultivated as a source of food for humans and animals [6].

Seaweed proteins, consisting of high-quality proteins superior to those found in most terrestrial plants, can be used to meet the essential amino acid (EAA) requirements for humans [7]. Red algae contain higher amounts of proteins [8,9], while brown algae contain lower amounts [10]. However, the main difference among the three groups of algae is the chemical composition and structural characteristics of the carbohydrates, which are highly variable and represent almost 50% of the dry matter [8,11]. In particular, in red algae, there is a high concentration of dietary fiber and polyphenols, giving them moderate digestibility [9].

Macroalgae have a low lipid content [7]; however, specifically in the case of red algae, essential fatty acids such as oleic, arachidonic, eicosapentaenoic, and docosahexaenoic acids are present, and the omega-6/omega-3 ratio is very low, which is highly beneficial for human health [9]. Although the fat content of seaweed is around 2% to 3% by dry weight, more than half of the fat is made up of unsaturated fatty acids, most of which are essential [12,13]. In general, the taxonomic location of seaweeds, as well as their growth conditions and seasonal and locality factors, can be associated with diversity in the amount and conformation of lipids and with fatty acid composition [2,14].

The compositions of amino acids, the derivatives of amino acids and peptides, carbohydrates, lipids, fatty acids, and sterols can be differentiated within the groups of macroalgae, demonstrating that their taxonomic classification is able to distinguish the species within a genus [15]. In particular, macroalgae lipids have been shown to have potential in the field of chemotaxonomy [16] because they distribute fatty acid methyl esters [17] and contain certain components such as fatty acids, sterols, and carotenoids, which have been found to be effective as taxonomic and phylogenetic markers [18,19]. Furthermore, fatty acid profiles are subgroup-specific and could serve as a chemotaxonomic tool for verifying the authenticity of seaweeds used as food [14,20].

Generally, the nutritional properties of seaweeds are estimated from their chemical compositions, but research regarding this information is not yet complete [21]. In Peru, very few studies have examined the chemical compositions and caloric contents of native edible algae, although data and methods that could be used for analysis are already available. To date, chemical–nutritional characterizations have been performed for a number of species of red algae, including *Mazzaella canaliculata* (=*Chondrus canaliculatus* [22]), *Rhodymenia corallina* (=*Rhodymenia howeana* [23]), *Chondracanthus chamissoi* [24,25,26], and *Porphyra columbina* [25], for the green alga *Ulva lactuca* [25], and for the brown alga *Macrocystis pyrifera* [26].

Recently, these commercially important species have undergone taxonomic changes as a result of phylogenetic studies. In the case of the “cochayuyo”, traditionally known under the name *P. columbina*, it is currently recognized within two genera, *Porphyra* and *Pyropia* [27]. For the group of “yuyos”, *C. chamissoi* and *Chondracanthus glomeratus*, a single genetic unit characterized by a variety of different forms has been suggested [28]. Recently, Roleda et al. [29] indicated the difficulty of differentiating commercially important taxa that are morphologically simple, such as the green algae known as “sea lettuce”, belonging to the *Ulva* genus, and the red algae known as “nori”, of the genera *Porphyra/Pyropia*, recommending that future publications require molecular identification at least for species that are cryptic and morphologically simple.

The seaweeds yuyo (*C. chamissoi*) and cochayuyo (*P. columbina* = *Porphyra*/*Pyropia*) were part of the diet of Peruvians in pre-Inca and Inca times, and even today, it is a tradition to consume them. Fresh yuyo is used mainly in the central and northern coastal areas, while the commercialization of dried cochayuyo is more common in central and southern Peru [30]. The harvest of cochayuyo is part of the seasonal migratory movements of the inhabitants of the mountains to the coast for agricultural work and artisanal fishing, in which the algae are part of the exchange of food and trade [31], according to Acleto [30]. Currently, cochayuyo is harvested, pressed into blocks to facilitate its transport, and sold in markets in different areas of the country. Furthermore, the demand for yuyo has increased and its price has risen by 400% due to the low availability of the alga [4] as it is an ingredient of ceviche, the best-known dish of the renowned Peruvian gastronomic tradition.

The recognition of the nutritional properties of edible macroalgae in Peru could increase their consumption in seaweed-based food preparations and strengthen their role in the country’s culinary heritage. Other species should be considered, such as *Callophyllis variegata* (“carola”), which has remarkable consumption characteristics (e.g., with respect to color, texture, flavor), and is sold to Asian markets (via Chile, 17 tons were sold in 2007 at a price of USD 25 per dry kilogram, according to the Instituto de Desarrollo Pesquero, IFOP) [32]. Additionally, present in the market is *M. canaliculata* (“alga flor”), offered as a food supplement. It should also be considered that the main uses of algae include the feeding of aquaculture species, dietary supplementation, and the formulation of diets that include algae in meals. It is important to increase the value of these resources and generate new products for consumption and commercialization, perhaps making it possible for some algae to be recommended as functional foods. In coastal Peru, seaweeds are an abundant and underexploited resource. Therefore, this work aimed to perform a comparative analysis of the chemical compositions of 37 samples of seaweeds (red, green, and brown) in order to determine their potential as food sources in the context of taxonomic changes.

## 2. Results and Discussion

This study of edible species from the Peruvian coast was carried out in the context of taxonomic changes. It included not only yuyos (*Chondracanthus*) and cochayuyos (*Porphyra*/*Pyropia*) but also other seaweeds that had the potential to be introduced into people’s diets and that, due to their morphological complexity, could be subjected to taxonomic scrutiny, as is the case for species of the genera *Ulva* and *Codium*. Thus, before the chemical analysis, phylogenetic and taxonomic information was considered. Additionally, the localities of origin of the samples were taken into account when addressing the challenge of delimiting the taxa. This study confirmed the relationships between algal groups on the basis of the chemical analysis.

In this study, 37 algal specimens (red, green, and brown) collected from different sampling localities on the Peruvian coast were biochemically analyzed to determine their potential as food. The following species stood out as particularly nutritional and showed potential for use in human diets: *C. chamissoi* and *Porphyra* spp./*Pyropia* spp. (known edible); the brown algae *Eisenia cokeri, Eisenia gracilis*, and *Lessonia berteroana*; the red algae *M. canaliculata, C. variegata*, and *R. corallina*; and the green algae *Ulva* sp. and *Codium* sp. The numbers of species belonging to the red and green algal genera currently under taxonomic revision are not specified.

### 2.1. Proximal Composition

Table 1 shows the percentages of protein, carbohydrate, lipid, ash, and moisture in the seaweed specimens collected in Peru. Among the red algae, Bangiales algae presented the highest average percentage of protein (24.10%), followed by the green algae from Ulvales with 18.73% (*p* = 0.000). There were no significant differences in the rest of the orders, for which values were in the range of 8.65–18.67%. In the case of the Laminariales brown algae of the genus *Eisenia*, the frond had a higher percentage of proteins (14.11%) than the stipe (9.46%) (*p* = 0.022). In general, these results agree with those of Thiviya et al. [33], in which red and green algae had a higher percentage of protein (8–47%) compared to brown algae (4–24%). However, in our study, the percentages in Bryopsidales green algae and Gigartinales red algae were no greater than those in the Laminariales brown algae. In particular, for red algae, the average protein percentage in Bangiales algae from Peru, from the genera *Porphyra* and *Pyropia*, were in the range of 21.09–28.91% and had similar reference values (21.2–32.71%) to those reported for *Porphyra* sp. from Japan, Korea, and China [34]; *Porphyra* sp. from New Zealand [35]; *P. columbina* from Argentina [36]; *Pyropia columbina* from Chile [37]; and *Porphyra* sp. and *Porphyra umbilicalis* from Portugal [38,39]. In the Gigartinales algae from Peru, *C. chamissoi,* and *M. canaliculata*, average protein concentrations of 12.02% and 11.25% were obtained, which are similar to the values obtained for *C. chamissoi* from Peru (12.16%) [26] and for *M. canaliculata* (=*Chondrus canaliculatus*) from Peru (12.48%) [22]. The protein percentage of *C. variegata* was lower (16.50%) in this study than that reported for *C. variegata* from southern Chile, which ranged from 20 to 28% [37,40]. In the case of *R. corallina* (Rhodymeniales), our results were half (14.58%) of those obtained by Rojas et al. [23] for *R. corallina* from Peru (28.56%, as *R. howeana*).

In green algae, the average protein percentage of the *Ulva* (Ulvales) algae from Peru in our study was in the range of 17.48–20.85%, which is within the range of reference values cited previously (8.65–27.2%) for *Ulva lactuca* from northern Chile [41], Iran [42], Norway [7], and Sweden [8]; for *Ulva stenophylla* from New Zealand [35]; and for *Ulva compressa* and *Ulva rigida* from Portugal [38,39]. In *Codium* sp. from Peru, our results showed a higher protein percentage (15.43%) than the average of 10.8% previously reported for *Codium fragile* from northern Chile [43]. In addition, for the brown algae Laminariales (*E. cokeri*, *E. gracilis,* and *L. berteroana*) in this study, the range of protein percentages was 8.65–16.12%, which is within the range of reference values obtained for the other Laminariales species, including *E. arborea* from Mexico, which had values between 5.5 and 11.7% [44], and *L. berteroana* from northern Chile, which had a value of 13.5% [45]. For *E. cokeri* and *E. gracilis* from Peru, significant differences were found in functional morphological structure when comparing the percentage of proteins in the frond with that in the stipe (14.11% vs. 9.45%). Similar observations were previously reported for *L. berteroana* from northern Chile [45].

The green algae showed the highest average lipid percentages. Bryopsidales showed the highest percentage (5.38%), followed by Ulvales (1.58%) (*p* = 0.000). The remaining orders of red and brown algae showed no significant differences, and their values ranged from 0.01 to 1.43%. The green seaweed *Codium* sp. (Bryopsidales) from Peru had a lipid percentage three times higher than that reported for *C. fragile* from northern Chile, which had a value of only 1.5% [41]. Additionally, for *Ulva* sp. (Ulvales) from Peru, the average lipid percentage was within the reference values (0.3–3.6%) obtained for *U. lactuca* from northern Chile [41], Iran [42], and Norway [7] and for *U. stenophylla* from New Zealand [35]. For red algae, the average lipid percentage in *Porphyra* sp. (Bangiales) from Peru was 0.56%, which is similar to the range of 0.25–1.00% reported for *Porphyra* sp. from China [34] and for *Porphyra* sp. from Argentina [36] but lower than the range of 2.0–2.8% reported for *Porphyra* sp. from Japan and Korea [34] and for *Porphyra* sp. from New Zealand [35]. In brown algae, the average lipid content of Laminariales from Peru was 0.60%, which is similar to the range given for the fronds of *Eisenia arborea* from Mexico [44] of 0.45–0.66 and for the fronds and stipes of *L. berteroana* from Chile [45] of 0.7–1.3%.

As for carbohydrates, according to the Kruskal–Wallis test, the red macroalgae Bangiales (59.85%) and Gigartinales (58.73%, *p* = 0.029) presented the highest medians, followed by the rest of the orders, with percentages ranging from 50.23 to 53.87%. For the Bangiales, our results were similar to the range of 39.85–60.36% reported for *Porphyra vietnamensis* from India [46]. In the case of Gigartinales, similar results were previously reported for *C. chamissoi* (62.65%) [26] and for *M. canaliculata* (65.06%) [22] from Peru. Additionally, for green algae, our results in *Ulva* sp. and *Codium* sp. were lower than those reported for *Ulva lactuca* from Iran (59.1%) [42] and from Chile (61.5%) [41] and for *Codium fragile* (66.8%) from Chile [43]. In the case of brown algae, the range of 43.3–54.3% reported for *Eisenia arborea* from Mexico [44] was similar to our results in *Eisenia* from Peru.

Regarding the percentage of ash, according to the Kruskal–Wallis test, the Bangiales red algae from Peru presented the lowest median (7.95%, *p* = 0.000) among other orders of red, green, and brown algae, which had values in the range of 9.50–36.45%. The red algae *Porphyra* sp. and *Pyropia* sp. (Bangiales) from Peru had a range similar to that of *P. columbina* from Argentina (average 6.46%) [36] and *Pyropia vietnamensis* (3.85–7.40%) [46]. However, previous results for *Porphyra* spp. from New Zealand [35] and for *Py. columbina* from Chile [37] were higher than our results, with average percentages between 15.1 and 19.8% for *Porphyra* sp. and *Py. columbina*. For green algae, our results for *Ulva* sp. were 11.0–32.2%, which are within the range established for *U. lactuca* from northern Chile [41], Iran [42], Norway [7], and Sweden [8] and for *U. stenophylla* from New Zealand [35]. For the brown algae *L. berteroana*, *E. cokeri*, and *E. gracilis* (Laminariales), a range of 9.50–36.45% was obtained for ash (%). These results were within the ranges from the references of 25.5–30.9 for the fronds of *E. arborea* from Mexico [44] and of 19.3–29.3% for the stipes and fronds of *L. berteroana* from Chile [45].

The moisture contents of all species ranged from 4 to 16%, and no relevant differences between the orders were demonstrated.

For species from Peru, it is necessary not only to introduce information regarding the chemical compositions of these edible seaweeds but also to discuss the species that could be included in the human diet because of their nutritional content. Leandro et al. [47] stated that most seaweeds are edible and can provide the macro- and micronutrients necessary for good nutrition. According to the dietary reference intakes (DRI), the total caloric intake in a balanced adult diet should be 10–35% protein, 46%–65% carbohydrates, and 20–35% lipids [48]. In this sense, seaweeds could represent an alternative to the animal products and legumes used as typical protein sources [47]. Our results agree with the protein DRI requirements as the Bangiales red algae from Peru had a percentage of 21.09–28.91%, followed by the Ulvales green algae, which was in the range of 17.48–20.85%. On one hand, seaweeds have high amounts of carbohydrates (in some species, carbohydrates represent more than 50% of the dry weight) [11]. According to our results, most of the analyzed samples had excellent carbohydrates content, which were similar to the values reported by DRI, especially the Bangiales (59.85%) and Gigartinales (58.73%) from Peru. On the other hand, seaweeds are known to be foods with low lipid contents [7]. At the order level, we found that only the Bryopsidales green algae from Peru had a considerable amount of lipids 5.38%. 

Finally, in terms of proximal composition, the seaweeds analyzed in this study are suitable for human consumption. Véliz et al. [49] stated that the flours made from 11 species of seaweeds from Chile possessed chemical compositions suitable for use as ingredients in human and animal diets, including *Ulva* sp. for green algae; *Durvillaea incurvata*, *Lessonia spicata*, *L. berteroana*, *Lessonia trabeculata*, and *M. pyrifera* for brown algae; and *Gracilaria chilensis*, *C. chamissoi*, *Cryptonemia obovata*, *Sarcodiotheca gaudichaudii,* and *Acrosorium* sp. for red algae.

### 2.2. Amino Acid Profile

The amino acid compositions of the lyophilized brown, red, and green algae are shown in Table 2, Table 3 and Table 4, respectively. The amino acids exhibiting the highest average concentrations were glutamic acid (2.17 g/100 g) and aspartic acid (2.05 g/100 g), followed by alanine (1.54 g/100 g) and the essential amino acid leucine (1.35 g/100 g), while those with the lowest concentrations were tyrosine (0.52 g/100 g), methionine (0.30 g/100 g), and histidine (0.30 g/100 g, *p* = 0.000). When analyzed by order, we found that green algae from Ulvales and red algae from Rhodymeniales and Bangiales presented the highest average concentrations of amino acids, with 1.34, 1.34, and 1.27 g/100 g, respectively, while the brown algae from Laminariales had the lowest average concentration (0.66 g/100 g, *p* = 0.000).

**Figure 1:**
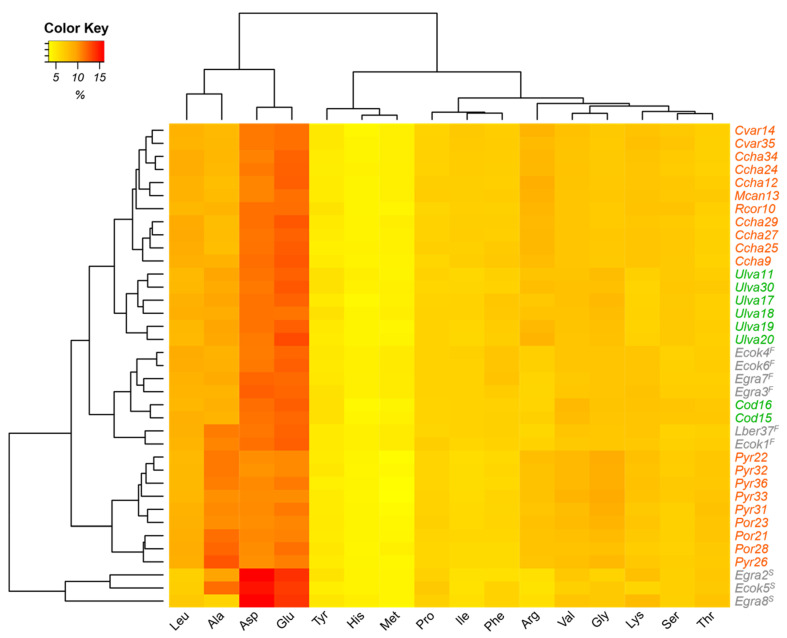
Heat map for classification of red, green, and brown macroalgae characterized by amino acid composition. The amino acid concentrations are expressed as relative percentages. *^F^* Frond; *^S^* stipe.

Furthermore, as can be seen from the heat map (Figure 1), a distinction within the algal groups based on amino acid composition was observed. In the case of red algae, there were two groups. The first group, the upper group, had higher concentrations of glutamic acid and aspartic acid; a predominance of the genera *Callophyllis, Mazzaella, Chondracanthus*, and *Rhodymenia*; and an identical profile. The second group, the lower group, had a higher concentration of alanine; lower amounts of glutamic acid and aspartic acid; and, in it, the genera *Porphyra* and *Pyropia* were predominant. In green algae, *Ulva* formed a separate group from *Codium* specimens. The brown algae were grouped into two sets according to their morphological compartment, i.e., stipe or frond. *E. cokeri* and *E. gracilis* were analyzed as stipes and had high relative percentages of glutamic acid and aspartic acid, while the species corresponding to fronds, *Eisenia* and *Lessonia,* presented lower relative percentages of these amino acids. According to Hernández-Carmona et al. [44], the nonessential amino acids most present in seaweeds are glutamic acid, aspartic acid, and alanine, and the most frequently present essential amino acid is leucine [33]. Several studies have indicated higher concentrations of these amino acids in green algae, followed by red and, to a lesser extent, brown algae [9,10,23,39,41,50,51,52,53]. In particular, the large amounts of amino acids such as glutamic acid are responsible for the special salty or umami taste of various algae [54].

Regarding the genera studied, the high and low amounts of amino acids reported were similar to those reported by other authors for the green algae *Ulva fasciata* from Brazil [55]; *U. capensis*, *U. rigida,* and *U. lactuca* from South Africa [56]; *U. lactuca* from Norway [7]; *U. lactuca* and *Ulva intestinalis* from Norway [10]; *U. lactuca* from China [21]; *U. rigida* from Portugal [39]; and *Codium decorticatum*, *C. spongiosum*, and *C. taylorii* from Brazil [55]. They were also similar to those reported for species of red algae including *Porphyra* sp. from Japan [57]; *P. acanthophora* from Brazil [55]; *Porphyra* sp. from Japan, Korea, and China [34]; *P. columbina* from Argentina [36]; *P. dioica*, *P. purpurea*, and *P. umbilicalis* from Norway [10]; *Porphyra* spp. from Portugal [58]; *P. umbilicalis* from Portugal [39]; and *C*. *chamissoi* from southern Peru [26]. With respect to brown algae, *E. cokeri and E. gracilis* from Peru presented the same ranges as were reported for *E. arborea* from Mexico [44] for glutamic acid (0.55–4.27 g/100 g), aspartic acid (0.40–3.67 g/100 g), alanine (0.22–4.30 g/100 g), and leucine (0.29–3.89 g/100 g).

Concerning the essential amino acids ratio (EAA%), the highest average was found in red algae from Gigartinales, with 48.65% (*p* = 0.000). The other orders did not show significant differences among them, with averages being between 43.71 and 45.98%. These values are higher than those reported for soy protein (39%) and very close to those for egg protein (47%) [59]. In addition, our results show that the red alga *C. chamissoi* collected from different localities in Peru had a higher amount of EAA% (average 48.41%) than that reported for *C. chamissoi* collected in Moquegua, in southern Peru (31.07%) [26]. Similarly, *Porphyra* sp./*Pyropia* sp. in our study had an EAA% of 44.48–46.65%, while the published values are in the range of 31.07–44.44% for *Porphyra* sp. from Japan, Korea, and China [34]; for *Porphyra* spp. from Portugal [58]; and for *P. umbilicalis* from Portugal [39]. This was the same for the green alga *Ulva* sp., which in our study had an EAA% of 44.54–46.22%, which is higher than the range determined (40.30–40.79%) for *U. lactuca* from Norway [7] and for *U. rigida* from Portugal [39].

Additionally, in terms of amino acid score (AAS), considering all of the taxa analyzed, the essential amino acids with the highest average scores were lysine, threonine, valine, and leucine (AAS > 0.96), followed by phenylalanine + tyrosine (0.91), and finally, isoleucine and histidine (0.82 > AAS > 0.80, *p* = 0.000). Numerically, the lowest average AAS was that of histidine from the Bryopsidales green algae (0.65). The latter result is in agreement with data previously reported in studies suggesting that sulfur-containing amino acids such as histidine are the major limiting amino acids in the proteins of some seaweeds [60].

Regarding the essential amino acid index (EAAI), the highest average percentages corresponded to Ulvales, Laminariales, Gigartinales, and Rhodymeniales (EAAI > 0.92), while the lowest percentages corresponded to Bryopsidales and Bangiales (0.90 > EAAI > 0.88, *p* = 0.000). Furthermore, in our study, the EAAI of *Porphyra* sp./*Pyropia* sp. was in the range of 0.87–0.90, which is slightly lower than the results obtained for *Porphyra* sp. from Japan, Korea, and China [34], and for *P. umbilicalis* and *P. dioica* from Portugal [39] was between 0.89 and 0.96, which is in agreement with those authors who have stated that the limiting essential amino acids are methionine, tryptophan, leucine, and isoleucine. Additionally, our EAAI results for the green alga *Ulva* sp. were in the range of 0.91–0.95, which is similar to the value reported by other authors indicating an EAAI between 0.92 and 1.23, with methionine as the limiting essential amino acid, in *U. lactuca* from Norway [7] and *U. rigida* from Portugal [39]. According to our results, most of the analyzed samples presented high and moderate quality with an EAAI value of >0.80 [61,62,63]. In addition, they had excellent profiles, with EAAI near that of egg protein and AASs higher than those of other plant proteins [33].

### 2.3. Fatty Acid Composition

Table 5, Table 6 and Table 7 show the fatty acid compositions of brown, red, and green algae. The highest average content of fatty acids corresponded to polyunsaturated fatty acids (PUFAs, 44.06%), followed by saturated fatty acids (SFAs, 34.56%), and finally monounsaturated fatty acids (MUFAs, 21.17%) (*p* = 0.000). The percentages of PUFAs in the references consulted for all seaweed groups were in the range of 30–60% [7,16,34,37,43]. However, values below 22% were also found [26,41,50,64]. The PUFAs found in the highest proportion were linolenic acid (C18:3n − 3) in the green algae group, arachidonic acid (ARA, C20:4n − 6) in the brown and red algae groups, and EPA (C20:5n − 3) in the red algae group.

In addition, a significant difference was observed in the MUFAs when orders were compared, with the stipe of the brown algae *Eisenia* (Laminariales) having the highest median (31.27%), while red algae from Bangiales had the lowest (11.40%) (*p* = 0.006). Previous studies have reported that Bangiales had a range of 14–21% [34,37,64], which is higher than our results. The MUFA present in the highest quantity was oleic acid (C18:1n − 9) in the brown algae from Laminariales and in the red algae from Gigartinales and Rhodymeniales.

As for SFAs, the fatty acid present in the highest quantity was palmitic acid (C16:0) for the three groups of algae, with the highest content being found in the red algae *C. chamissoi* (Gigartinales), with a median of 46.98%, and the lowest in the frond of the brown algae *Eisenia* (Laminariales), with 25.51%. These results agree with those reported by Sohrabipour et al. [65], wherein palmitic acid was the fatty acid with the highest amount, regardless of the species of seaweed studied.

**Figure 2:**
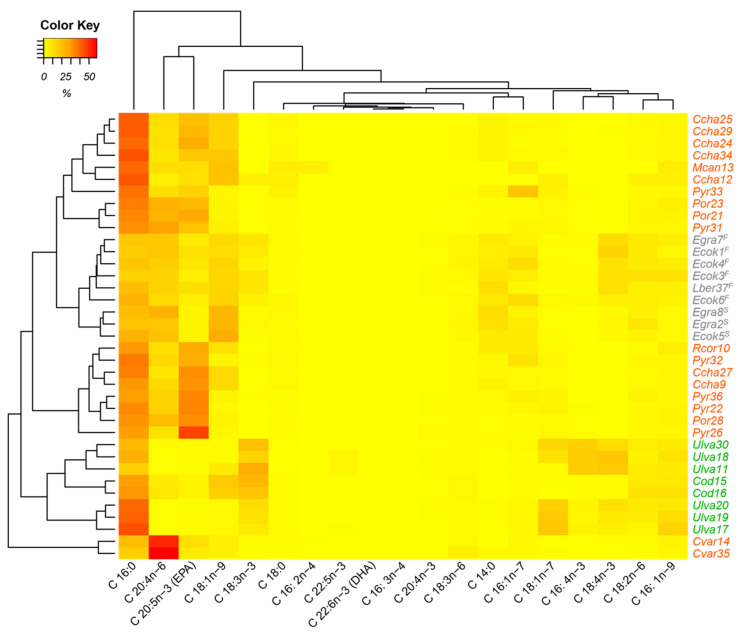
Heat map of classification of red, green, and brown macroalgae from the Peruvian coast characterized by fatty acid composition. Fatty acid concentrations are expressed as relative percentages. *^F^* Frond; *^S^* stipe.

In the heat map (Figure 2), the three groups of algae are organized according to the evaluated composition of the fatty acids. There are two clearly separate groups. One belongs to the green algae, which have a cluster related to C18:3n − 3 acid. The brown algae form another cluster, which is related to the fatty acids C20:4n − 6 and C18:1n − 9, whereby a higher percentage of the C18:1n − 9 fatty acid is presented in the stipe. This allows separation from the frond. Elsewhere, red algae form three clusters with three discriminating fatty acids, C16:0, EPA, and C20:4n − 6. The top cluster, formed by *C. chamissoi* and *M. canaliculata* (Gigartinales) and *Pyropia* and *Porphyra* (Bangiales), is distinguished by its higher percentage of C16:0; the central cluster, consisting of the red algae *C. chamissoi* (Gigartinales), *Pyropia* and *Porphyra* (Bangiales), and *R. corallina* (Rhodymeniales), presents higher percentages of EPA; and the lowest cluster, formed by the red alga *C. variegata* (Gigartinales), has a higher percentage of C20:4n − 6. The characteristic fatty acids found in the heat map for the algal clusters have also been reported in different publications: Green algae from Ulvales and Bryopsidales had a significant relative percentage of C18:3n − 3 between 7 and 27% for *U. lactuca* from the USA [66] and Norway [7]; for *Ulva* sp. from southern Chile [67]; for *Codium fragile* from northern Chile [43]; and for *C. tomentosum* from Portugal [68]. Similarly, brown algae from Laminariales present significant relative percentages of C20:4n − 6 (9–34%) and C18:1n − 9 (9–17%) for *Lessonia flavicans* from southern Chile [67]. Finally, red algae from Gigartinales and Bangiales have significant relative percentages of C16:0 (30–64%), EPA (6–42%), and C20:4n − 6 (1–17%) for *P. tenera* from Japan [57]; for *Porphyra* sp. from Japan, Korea, and China [34]; for *C. variegata* from Chile [37]; for *P. columbina* from Argentina [36]; for *M. canaliculata* from the central coast of Peru [22]; and for *Py. columbina* from southern Chile [67].

With respect to omega-6 content (ω6), arachidonic acid (C20:4n − 6) was the compound with the highest amount, with 54.73% for the red alga *C. variegata* (Gigartinales), followed by the stipe (20. 06%) and the frond (15.21%) of *Eisenia* (Laminariales) and Bangiales (14.13%). The lowest median of C20:4n − 6 was found for the green algae from Bryopsidales (6.56%) and Ulvales (0.37%) (*p* = 0.001). The relative percentage of ω6 for *C. variegata* from Peru (58.81%) was three times higher than the value reported for *C. variegata* from southern Chile, which was 19.15% [37]. In the case of the Laminariales algae studied, *L. berteroana*, *E. cokeri*, and *E. gracilis*, our results (18.03–31.04%) were similar to those reported for *L. flavicans* from southern Chile, with a relative percentage between 15 and 43% [67]. The green algae of the genus *Ulva* had a relative percentage of ω6 between 3.61 and 8.19%, which is similar to the reference values (3 and 12%) for *U. lactuca* from the USA [66], northern Chile [41], and Norway [7]; *U. armoricana* from France [16]; and *Ulva* sp. from southern Chile [67].

In terms of omega-3 (ω3) content, at the group level, using the Kruskal–Wallis test, the main fatty acids were found to be EPA (C20:5n − 3) in red algae, with a median of 24.19%, and C18:3n − 3 in green algae, with a median of 16.11%. In brown algae, EPA (C20:5n − 3) (5.28%) and stearidonic acid (C18:4n − 3) (8.73%) were important. Fatty acid ω3 content showed no significant differences when comparing orders (*p* = 0.180).

At the order level, using the Kruskal–Wallis test, the highest median ω6/ω3 ratio was found in the Gigartinales red alga *C. variegata* (6.59), followed by stipes of *Eisenia* (Laminariales) (3.78). For the rest of the algae, the ratio did not exceed the median value of 1 (*p* = 0.001). The value of the ω6/ω3 ratio for the alga *C. variegata* from the central coast of Peru was remarkable, with none of the references consulted approaching it in value. The ω6/ω3 ratios reported for the orders Ulvales, Bangiales, and Laminariales from southern Chile [67] had the same tendencies, as shown in our results; the Laminariales had a ratio higher than 1, and the rest of the orders had a ratio lower than 1. Healthy diets have ω6/ω3 ratios with values lower than 10 [69] or with a maximum of 4 [70,71] since higher ratios favor the risk of developing diseases [72]. Based on these results, the red alga *C. variegata* from two localities in Peru (Paracas and Marcona in Ica) could be used in food because of its high content of ω6, which increases the ratio ω6/ω3.

This study showed that Bangiales (cochayuyos; *Porphyra* sp. and *Pyropia* sp.) had the highest average percentage of protein and carbohydrates and the lowest values of ash. Additionally, Ulvales specimens (sea lettuce, *Ulva* sp.) were remarkable because of their protein content, and Bryopsidales (*Codium* sp.) were remarkable because of their lipid contents. The highest amounts of essential amino acids were found in Gigartinales (yuyo, *C. chamissoi*; alga flor, *M. canaliculata*; and carola, *C. variegata*), while the highest essential amino acid index score was found for Ulvales (*Ulva* sp.), Laminariales (“kelps” *E. cokeri, E. gracilis*, and *L. berteroana*), Gigartinales (*C. chamissoi*, *M. canaliculata,* and *C. variegata*), and Rhodymeniales (*R. corallina*). The major PUFAs were linolenic acid in the group of green algae, arachidonic acid in the group of brown and red algae, and EPA in the group of red algae. A major MUFA was oleic acid in Laminariales, Gigartinales, and Rhodymeniales. With respect to the SFAs, palmitic acid had the highest quantity in the Gigartinales specimens. With regard to the content of omega-6, ARA was predominantly found in in the red alga *C. variegata* (Gigartinales), while for omega-3, it was EPA in red and green algae. 

Knowledge regarding edible algae is scarce in Peru, so it was considered necessary to provide detailed and complete information on the chemical compositions of seaweeds that are consumed and that have been consumed since pre-Inca times in Peru, such as yuyos and cochayuyos (red algae), as well as to include data on other potentially edible species. From here, it is possible to revalue these edible algae and emphasize the consumption of other groups (orders) of seaweeds that are nutritionally promising, such as green and brown algae, which are not commonly consumed but could be introduced into the diet not only as a protein source but also for their important contents of essential amino acids, polyunsaturated fatty acids, and carbohydrates. These findings provide, for the first time in Peru, relevant information on the chemical and nutritional composition of seaweeds that could potentially be used directly as food or as ingredients in human or animal diets. It is also a first step in connecting the differences and similarities in the chemical compounds of these taxa with their taxonomic positions since it is important to understand these entities at a specific level in order for them to be commercialized internally or externally. Despite the amount of information generated, the grouping of the different taxa did not reflect a well-defined taxonomic classification, although it allowed the chemical characterization of the edible species analyzed. With that, we reinforce the definition of integrative taxonomy [73], in which species should be revised, analyzing different characteristics, using other methods, and applying other criteria to delimit species.

## 3. Materials and Methods

### 3.1. Sample Collection and Preparation

**Figure 3:**
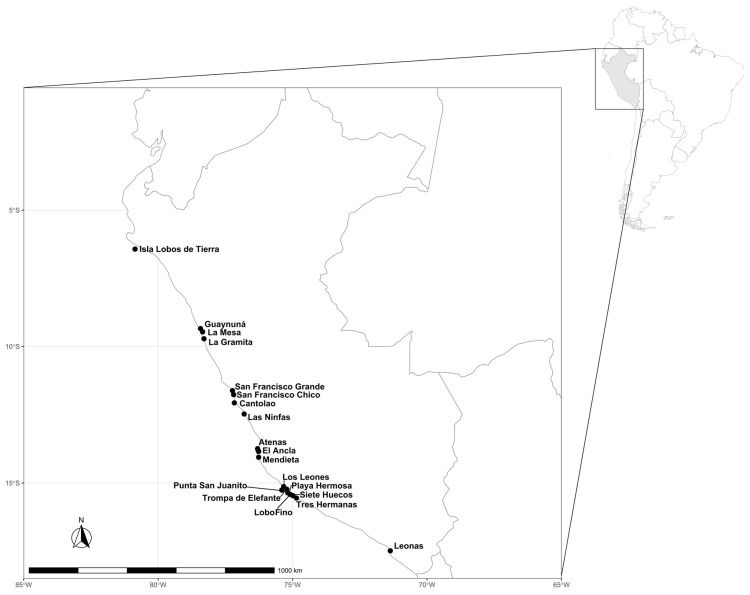
Map of the Peruvian coast showing the collection localities of edible seaweeds.

During 2018 and 2019, algal samples were collected and identified in the rocky intertidal and subtidal zone along the Peruvian coast (6°–17° S): Lobos de Tierra Island (Piura), Casma (Ancash), Ancón, Callao and Pucusana (Lima), Paracas and San Juan de Marcona (Ica), and Ilo (Moquegua) (Table 8). A total of 37 specimens were analyzed, belonging to the 3 groups of seaweed (Table 1)—21 Rhodophyta (red), 8 Chlorophyta (green), and 9 Ochrophyta-Phaeophyceae (brown)—and were collected from 19 localities on the Peruvian coast (6°–17° S) (Figure 3). Among the red algae, there were six genera, four of which were identified at the species level (*C. variegata, M. canaliculata, C. chamissoi*, and *R. corallina*), while for *Porphyra* and *Pyropia* the species were not determined. Among the green algae, *Ulva* and *Codium* were identified at the generic level. With respect to brown algae, three species, *E. cokeri, E. gracilis*, and *L. berteroana*, were identified. In the kelp *Eisenia* (Laminariales) alone, two different parts of the thallus were analyzed, the stipe and the frond. 

Fresh material was selected and transported to IMARPE (Marine Institute of Peru), hermetically sealed in plastic bags, and kept in refrigerated containers. Epiphytes and contaminants were removed from the collected material via repeated washing in fresh water. The cleaned algae were then frozen at −20 °C. Drying was performed in a Labconco freeze-dryer, model 18 L (Kansas, KS, USA) [74]. The conditions were as follows: 0.022 and 0.070 mbar (vacuum pressure), −56 °C (collection temperature), and −15 °C for 8 h, followed by an increase of 0.5 °C/min up to 5 °C for 15 h, and a further increase of 0.5 °C/min up to 25 °C for 7 h (ramp temperature). Finally, pulverized lyophilized sample was obtained using a mortar and stored in hermetically sealed plastic bags at −20 °C.

### 3.2. Proximate Analysis

To measure the moisture percentage, 0.1 g of lyophilized sample was weighed and dried in a Vacucell vacuum oven (MMM, Germany) at 105 °C for 16 h at a pressure below 0.1 bar [75]. Additionally, to analyze the ashes, 0.1 g of lyophilized sample was weighed and incinerated in a Thermolyne muffle furnace (F6010, USA) at 550 °C for 16 h [76].

Proteins were quantified from 5 mg of lyophilized sample using the Hartree method [77]. Hydrolysis was carried out with 5 mL of 0.5 N sodium hydroxide at 85 °C for 45 min. A solution containing Folin–Ciocalteu’s reagent, sodium carbonate, copper sulfate, and potassium and sodium tartrate was then added. The absorbance was measured at 650 nm using a UV spectrophotometer (Varian, Australia), and the percentage of proteins was measured using a calibration curve with bovine serum albumin (300 μg/mL) in a concentration range of 0–150 μg/mL.

The modified Folch method was used for the analysis of lipids [78]. Lipids were extracted from 25 mg of lyophilized seaweed with 3 mL of a chloroform–methanol (2:1) solvent mixture. The extraction was performed twice in a sonicator at 4 °C for 30 min, following which 2 mL of 0.88% (*w*/*v*) potassium chloride was added. The organic phase was dried in an evaporator–concentrator with nitrogen gas. The lipid extracts were stored under vacuum and in the dark for 14 h and then weighed to obtain the percentage of total lipids.

Carbohydrates were quantified using the following equation: Carbohydrate% = 100% − Protein% − Lipid% − Ash% − Moisture% [22,35,37].

Proximate assays were performed in triplicate.

### 3.3. Fatty Acid Analysis

The determination of fatty acids was performed by adding toluene, methanol, and hydrochloric acid to 1 mg of the lipids and 0.1 mg of methyl tricosanoate (C23:0, internal standard) and incubating the mixture at 45 °C for 14 h for the derivatization [79]. The produced methylesters were extracted with hexane for gas chromatography analysis on a Varian GC-FID CP-3800 (Germany) under the following conditions: 30 m × 0.25 mm × 0.25 µm fused silica Restek Stabilwax^®^ WCOT column; splitless injection method (0.50 min); injection volume of 1 μL; injector temperature at 250 °C; helium carrier gas at a flow rate of 1 mL/min; ramp 120 °C per minute; 30 °C/min to 160 °C; 160 °C per minute; 4 °C/min to 240 °C; 240 °C for 7 min. The total duration of the temperature program was between 30 and 33 min, and the programmed temperature of detection was 260 °C. Fatty acids assays were performed in triplicate.

### 3.4. Amino Acid Analysis

Between 30 and 50 mg of lyophilized sample was hydrolyzed with 2 mL of 6 N HCl at 112 °C for 24 h [80]. Next, following the AccQ-Fluor reagent kit manual (Waters Corporation, Milford, MA, USA), a solution containing 50 µL of the hydrolyzed sample, 100 µL of the 2.5 mM 2-L-aminobutyric acid internal standard, and 4850 µL of ultrapure water was prepared, filtered on 0.45 µm PTFE filters, and derivatized. After that, 5 µL of the derivatized amino acid solution was injected in the HPLC-FL Elite LaChrom (Hitachi, Japan). The equipment conditions were as follows: Hypersil GOLD C18 column 5 µm × 4.6 mm × 150 mm; mobile phase flow rate 1 mL/min at 37 °C (column); 250 nm (excitation); 395 nm (emission); 5 µL (injection); mobile phase A: ultrapure water; mobile phase B: sodium acetate pH 5.1; mobile phase C: acetonitrile; one mobile phase ramp: 0 min (0, 100, 0), 1 min (0, 100, 0), 25 min (0, 83, 27), 33 min (50, 0, 50), 36 min (0, 100, 0), and 40 min (0, 100, 0). The amino acids were quantified by a calibration curve with a concentration ranging from 5 to 50 pmol/µL. The amino acids identified were alanine, arginine, aspartic acid, glutamic acid, glycine histidine, isoleucine, leucine, lysine, methionine, phenylalanine, proline, serine, threonine, tyrosine, and valine (Ala, Arg, Asp, Glu, Gly, His, Ile, Leu, Lys, Met, Phe, Pro, Ser, Thr, Tyr, and Val). The applied analytical technique was not able to identify the amino acids cysteine and tryptophan (Cys, Trp). Amino acids assays were performed in triplicate.

### 3.5. Essential Amino Acid Index (EAAI)

The amino acid score (AAS*i*) was calculated for each essential amino acid *i* as the quotient of the relative percentage of the essential amino acid *i* in the sample to the relative percentage of amino acid *i* in relation to a standard protein [81,82]. In this study, egg protein was used as the standard protein in accordance with FAO [59], which is applicable for measuring protein quality in human nutrition. Chemical scores greater than 1 were replaced with 1. The proposed essential amino acid index is presented as Equation (1) [82].

(1) $$EAAI=\sqrt[{n}]{{AAS}_{1}\times {AAS}_{2}\ldots \times {AAS}_{n}}$$

Moreover, the essential amino acids ratio (EAA%) was determined as the sum of each essential amino acid among the total amino acids.

### 3.6. Statistical Analysis

Comparisons of the concentrations of biochemical compounds were made at the taxonomic level of order. For this, analysis of variance (ANOVA) was used at a significance level of α = 0.05, followed by Tukey’s post hoc test. If the ANOVA assumptions were not met, the Kruskal–Wallis test was used. The multivariate analysis was performed using a heat map to obtain the relationship between the concentrations of biochemical compounds and the analyzed seaweed species. The data were analyzed using the statistical programs Minitab 19 and the package heatmap.2 of R-studio v 1.2.1335.

## Figures and Tables

**Table 1:** Proximate composition of lyophilized Peruvian seaweeds.

	Order	Code	Protein (%)	Carbohydrate (%)	Lipid (%)	Ash (%)	Moisture (%)
Brown Algae	*Laminariales*	*Ecok1 ^F^*	11.50 ± 0.09	46.99 ± 0.23	1.43 ± 0.04	30.18 ± 0.25	9.90 ± 0.11
		*Egra3 ^F^*	15.34 ± 0.08	60.19 ± 0.65	0.36 ± 0.06	10.56 ± 0.28	13.55 ± 1.07
		*Ecok4 ^F^*	16.12 ± 0.05	59.11 ± 1.42	0.91 ± 0.06	9.50 ± 0.00	14.36 ± 1.53
		*Ecok6 ^F^*	14.40 ± 0.23	60.88 ± 0.38	0.50 ± 0.03	11.58 ± 0.03	12.64 ± 0.09
		*Egra7 ^F^*	13.18 ± 0.90	48.45 ± 0.43	0.46 ± 0.14	27.22 ± 0.33	10.69 ± 0.66
		mean	14.11 ^y^	54.31	0.73	17.81	12.23
		*Ecok5 ^S^*	4.81 ± 0.13 *	49.54 ± 0.09	0.14 ± 0.03	36.45 ± 0.36	9.06 ± 0.34
		*Egra2 ^S^*	8.78 ± 0.32	50.20 ± 0.62	0.36 ± 0.05	30.32 ± 0.71	10.34 ± 0.35
		*Egra8 ^S^*	10.13 ± 0.39	50.93 ± 0.33	0.36 ± 0.06	25.52 ± 0.38	13.06 ± 1.04
		mean	9.45 ^z^	50.22	0.29	30.76	10.82
		*Lber37 ^F^*	8.65 ± 0.22	50.23 ± 0.44	0.85 ± 0.17	27.38 ± 0.14	12.89 ± 0.09
	mean or median		12.26 ^c^	50.23	0.60 ^c^	27.22	12.64
Red Algae	*Gigartinales*	*Ccha12*	11.32 ± 0.21	62.25 ± 1.16	0.01 ± 0.10	18.19 ± 0.21	8.23 ± 1.06
		*Ccha24*	10.29 ± 0.15	59.28 ± 0.24	0.53 ± 0.02	20.83 ± 0.13	9.06 ± 0.21
		*Ccha27*	14.54 ± 0.42	58.48 ± 0.14	0.29 ± 0.10	16.65 ± 0.44	10.04 ± 0.06
		*Ccha29*	15.59 ± 0.46	56.49 ± 0.17	0.31 ± 0.07	17.73 ± 0.56	9.88 ± 0.20
		*Ccha34*	10.36 ± 0.59	58.97 ± 0.38	0.18 ± 0.09	19.82 ± 0.21	10.66 ± 0.09
		*Ccha9*	10.52 ± 0.49	55.02 ± 0.78	0.28 ± 0.23	20.41 ± 0.01	13.76 ± 0.08
		*Ccha25*	11.50 ± 0.87	57.36 ± 1.20	0.26 ± 0.22	20.53 ± 0.36	10.35 ± 0.19
		*Cvar14*	14.34 ± 0.15	59.18 ± 0.50	0.75 ± 0.04	19.90 ± 0.29	5.84 ± 0.02
		*Cvar35*	18.67 ± 1.21	52.48 ± 0.15	1.24 ± 0.39	19.36 ± 0.04	8.24 ± 0.72
		*Mcan13*	11.25 ± 1.47	63.05 ± 1.44	0.02 ± 0.00	17.86 ± 0.09	7.81 ± 0.12
	mean or median		12.84 ^c^	58.73	0.39 ^c^	19.59	9.47
	*Bangiales*	*Por21*	27.40 ± 0.69	52.87 ± 1.39	0.72 ± 0.06	8.49 ± 0.08	10.52 ± 0.68
		*Por23*	27.21 ± 0.82	58.74 ± 0.73	0.73 ± 0.05	7.09 ± 0.25	6.23 ± 0.11
		*Por28*	23.09 ± 0.10	56.48 ± 0.61	0.33 ± 0.15	7.64 ± 0.12	6.86 ± 0.90
		*Pyr22*	23.95 ± 0.68	59.85 ± 0.90	0.39 ± 0.01	7.95 ± 0.39	7.86 ± 0.16
		*Pyr26*	28.91 ± 1.15	62.08 ± 1.03	0.87 ± 0.11	6.87 ± 0.46	6.86 ± 0.03
		*Pyr31*	21.09 ± 0.67	64.64 ± 1.16	0.54 ± 0.00	9.01 ± 0.23	4.72 ± 0.26
		*Pyr32*	19.02 ± 0.75	59.18 ± 0.83	0.41 ± 0.15	9.07 ± 0.10	12.31 ± 0.13
		*Pyr33*	16.71 ± 1.40 *	62.03 ± 1.75	0.36 ± 0.14	8.19 ± 0.19	12.71 ± 0.30
		*Pyr36*	22.16 ± 1.26	62.70 ± 0.58	0.65 ± 0.14	6.96 ± 0.28	7.53 ± 0.27
	mean or median		24.10 ^a^	59.85 ^†^	0.56 ^c^	7.95 ^††^	7.53
	*Rhodymeniales*	*Rcor10*	14.58 ± 0.10	52.52 ± 0.34	0.14 ± 0.03	20.44 ± 0.14	12.32 ± 0.26
Green Algae	*Bryopsidales*	*Cod15*	16.51 ± 1.05	58.11 ± 1.46	5.92 ± 0.35	12.25 ± 0.17	7.22 ± 0.24
		*Cod16*	14.35 ± 0.25	42.81 ± 1.02	4.84 ± 0.50	30.82 ± 0.19	7.18 ± 0.08
	mean or median		15.43 ^bc^	50.46	5.38 ^a^	21.54	7.20
	*Ulvales*	*Ulva11*	17.48 ± 0.49	52.31 ± 0.92	1.51 ± 0.11	13.49 ± 0.14	15.21 ± 0.18
		*Ulva18*	18.58 ± 0.42	61.77 ± 0.10	2.39 ± 0.23	11.21 ± 0.17	6.05 ± 0.46
		*Ulva19*	17.56 ± 1.69	52.31 ± 2.52	1.08 ± 0.15	18.06 ± 0.86	10.99 ± 0.11
		*Ulva20*	20.09 ± 0.12	55.43 ± 2.57	1.85 ± 0.18	12.16 ± 0.50	9.38 ± 0.25
		*Ulva30*	17.82 ± 0.20	56.52 ± 0.45	1.74 ± 0.24	26.63 ± 0.09	7.82 ± 0.05
		*Ulva17*	20.85 ± 2.57	45.99 ± 0.11	0.90 ± 0.10	13.07 ± 0.14	9.75 ± 0.24
	mean or median		18.73 ^b^	53.87	1.58 ^b^	13.28	9.57

The results are means of triplicate determination ± standard deviation. The comparisons of protein percentage by orders showed significant differences indicated by letters ^a–c^, according to the Tukey test. The same applies to the lipid percentage, where letters ^a–c^ indicate significant differences according to the Tukey test. In the case of letters y and z, they indicate significant differences when comparing the protein percentage of the frond and stipe of the Laminariales order. Means that do not share a **letter** are significantly different. Medians were compared by Kruskal–Wallis test for carbohydrates (%), ash (%), and moisture (%). *^F^* Frond; *^S^* stipe; * outliers; ^†^ *z*-value = 2.41 (*p* = 0.029); ^††^ *z*-value = −4.44 (*p* = 0.000).

**Table 2:** Amino acid concentration (g/100 g), essential amino acid ratio (EAA ratio), amino acid score (AAS), and essential amino acid index (EAAI) of lyophilized species of brown macroalgae.

	Laminariales									
	*Ecok1 ^F^*	*Egra3 ^F^*	*Ecok4 ^F^*	*Ecok6 ^F^*	*Ecok5 ^S^*	*Egra7 ^F^*	*Egra2 ^S^*	*Egra8 ^S^*	*Lber37 ^F^*	Mean
Essential amino acids										
Arginine	0.47 ± 0.01	0.72 ± 0.01	0.9 ± 0.08	0.74 ± 0.01	0.16 ± 0.01	0.55 ± 0.00	0.23 ± 0.01	0.29 ± 0.00	0.35 ± 0.01	0.49
Histidine	0.19 ± 0.00	0.31 ± 0.01	0.35 ± 0.02	0.29 ± 0.00	0.09 ± 0.00	0.24 ± 0.01	0.12 ± 0.00	0.17 ± 0.00	0.15 ± 0.00	0.21
Isoleucine	0.42 ± 0.02	0.79 ± 0.01	0.88 ± 0.07	0.72 ± 0.01	0.16 ± 0.01	0.57 ± 0.01	0.23 ± 0.01	0.29 ± 0.00	0.39 ± 0.00	0.50
Leucine	0.72 ± 0.03	1.24 ± 0.02	1.47 ± 0.11	1.21 ± 0.02	0.26 ± 0.02	0.94 ± 0.02	0.34 ± 0.01	0.45 ± 0.00	0.64 ± 0.00	0.81
Lysine	0.55 ± 0.02	1.02 ± 0.01	1.04 ± 0.08	0.87 ± 0.01	0.22 ± 0.02	0.72 ± 0.01	0.44 ± 0.00	0.55 ± 0.02	0.50 ± 0.00	0.66
Methionine	0.24 ± 0.00	0.39 ± 0.01	0.45 ± 0.02	0.38 ± 0.00	0.08 ± 0.00	0.29 ± 0.00	0.10 ± 0.00	0.12 ± 0.00	0.19 ± 0.00	0.25
Phenylalanine	0.46 ± 0.01	0.87 ± 0.09	1.06 ± 0.09	0.87 ± 0.01	0.24 ± 0.03	0.75 ± 0.01	0.24 ± 0.01	0.38 ± 0.00	0.42 ± 0.00	0.59
Threonine	0.52 ± 0.01	0.86 ± 0.02	0.94 ± 0.07	0.8 ± 0.01	0.28 ± 0.02	0.63 ± 0.01	0.4 ± 0.01	0.50 ± 0.01	0.41 ± 0.00	0.60
Valine	0.53 ± 0.03	0.93 ± 0.01	1.07 ± 0.09	0.89 ± 0.01	0.26 ± 0.01	0.67 ± 0.00	0.38 ± 0.01	0.48 ± 0.01	0.49 ± 0.00	0.63
Non-essential amino acids										
Alanine	1.03 ± 0.05	1.21 ± 0.01	1.36 ± 0.12	1.18 ± 0.00	0.61 ± 0.01	1.04 ± 0.04	0.62 ± 0.00	0.37 ± 0.52	0.91 ± 0.00	0.93
Aspartic acid	1.13 ± 0.04	2.05 ± 0.02	1.96 ± 0.13	1.68 ± 0.00	0.84 ± 0.01	1.50 ± 0.03	1.19 ± 0.01	1.40 ± 0.03	0.94 ± 0.00	1.41
Glutamic acid	1.21 ± 0.05	1.99 ± 0.03	2.17 ± 0.17	1.87 ± 0.01	0.73 ± 0.02	1.47 ± 0.02	1.02 ± 0.00	1.19 ± 0.02	1.02 ± 0.02	1.41
Glycine	0.55 ± 0.05	0.96 ± 0.03	1.07 ± 0.08	0.90 ± 0.00	0.28 ± 0.01	0.73 ± 0.02	0.31 ± 0.01	0.45 ± 0.00	0.48 ± 0.00	0.64
Proline	0.50 ± 0.01	0.79 ± 0.01	0.85 ± 0.07	0.73 ± 0.01	0.28 ± 0.02	0.58 ± 0.01	0.34 ± 0.00	0.40 ± 0.00	0.36 ± 0.01	0.54
Serine	0.48 ± 0.01	0.86 ± 0.03	0.87 ± 0.06	0.74 ± 0.00	0.26 ± 0.02	0.62 ± 0.01	0.34 ± 0.01	0.40 ± 0.00	0.40 ± 0.00	0.55
Tyrosine	0.25 ± 0.00	0.54 ± 0.02	0.49 ± 0.03	0.40 ± 0.00	0.10 ± 0.01	0.34 ± 0.00	0.22 ± 0.00	0.24 ± 0.02	0.21 ± 0.00	0.31
EAA ratio (%)	44.32	45.91	48.23	47.44	36.14	46.05	38.04	42.28	44.98	43.71
AAS										
Histidine	0.98	0.9	0.9	0.91	1	0.93	0.95	1	0.89	0.94
Isoleucine	0.79	0.83	0.82	0.81	0.7	0.8	0.66	0.64	0.84	0.77
Leucine	1	0.97	1	1	0.84	1	0.75	0.75	1	0.92
Lysine	1	1	1	1	1	1	1	1	1	1
Phenylalanine + Tyrosine	0.88	0.97	0.96	0.95	0.97	1	0.87	0.91	0.88	0.93
Threonine	1	1	1	1	1	1	1	1	1	1
Valine	0.92	0.89	0.92	0.92	0.99	0.87	1	0.99	0.96	0.94
EAAI	0.94	0.93	0.94	0.94	0.92	0.94	0.88	0.89	0.94	0.92

The results are means of triplicate determination ± standard deviation. *^F^* Frond; *^S^* stipe.

**Table 3:** Amino acid concentration (g/100 g), essential amino acid ratio (EAA ratio), amino acid score (AAS), and essential amino acid index (EAAI) of lyophilized species of red macroalgae.

	Gigartinales										
	*Ccha9*	*Ccha12*	*Ccha24*	*Ccha27*	*Ccha29*	*Ccha34*	*Ccha25*	*Cvar14*	*Cvar35*	*Mcan13*	Mean
Essential amino acids											
Arginine	0.85 ± 0.06	1.03 ± 0.01	0.88 ± 0.01	1.05 ± 0.00	1.13 ± 0.07	0.76 ± 0.00	0.90 ± 0.04	1.37 ± 0.00	1.44 ± 0.01	0.80 ± 0.01	1.08
Histidine	0.22 ± 0.02	0.21 ± 0.00	0.22 ± 0.00	0.25 ± 0.00	0.28 ± 0.02	0.17 ± 0.00	0.22 ± 0.01	0.25 ± 0.00	0.34 ± 0.00	0.17 ± 0.00	0.25
Isoleucine	0.65 ± 0.04	0.67 ± 0.01	0.66 ± 0.01	0.77 ± 0.00	0.83 ± 0.05	0.56 ± 0.01	0.63 ± 0.04	0.99 ± 0.01	1.14 ± 0.00	0.53 ± 0.01	0.79
Leucine	0.99 ± 0.07	0.99 ± 0.01	0.99 ± 0.00	1.25 ± 0.00	1.34 ± 0.07	0.85 ± 0.00	1.01 ± 0.06	1.39 ± 0.02	1.59 ± 0.00	0.79 ± 0.00	1.18
Lysine	0.74 ± 0.05	0.76 ± 0.01	0.75 ± 0.00	0.89 ± 0.00	0.93 ± 0.04	0.63 ± 0.01	0.75 ± 0.04	1.13 ± 0.02	1.35 ± 0.01	0.63 ± 0.00	0.92
Methionine	0.21 ± 0.01	0.25 ± 0.01	0.24 ± 0.01	0.27 ± 0.00	0.33 ± 0.02	0.21 ± 0.00	0.23 ± 0.01	0.36 ± 0.00	0.40 ± 0.00	0.20 ± 0.00	0.28
Phenylalanine	0.70 ± 0.05	0.66 ± 0.01	0.64 ± 0.00	0.75 ± 0.00	0.82 ± 0.04	0.55 ± 0.01	0.68 ± 0.04	0.96 ± 0.01	1.12 ± 0.00	0.56 ± 0.01	0.79
Threonine	0.60 ± 0.04	0.68 ± 0.00	0.60 ± 0.00	0.73 ± 0.00	0.78 ± 0.03	0.52 ± 0.00	0.63 ± 0.04	0.92 ± 0.01	1.04 ± 0.01	0.57 ± 0.00	0.75
Valine	0.73 ± 0.05	0.78 ± 0.01	0.76 ± 0.01	0.88 ± 0.00	0.93 ± 0.05	0.64 ± 0.00	0.73 ± 0.04	1.15 ± 0.01	1.31 ± 0.00	0.63 ± 0.01	0.91
Non-essential amino acids											
Alanine	0.97 ± 0.06	0.85 ± 0.01	0.87 ± 0.00	0.99 ± 0.00	1.06 ± 0.02	0.76 ± 0.00	0.82 ± 0.05	1.25 ± 0.02	1.49 ± 0.00	0.71 ± 0.01	1.06
Aspartic acid	1.48 ± 0.08	1.37 ± 0.03	1.38 ± 0.01	1.68 ± 0.00	1.86 ± 0.07	1.11 ± 0.01	1.44 ± 0.07	2.02 ± 0.02	2.36 ± 0.00	1.11 ± 0.01	1.71
Glutamic acid	1.62 ± 0.10	1.62 ± 0.04	1.53 ± 0.01	1.82 ± 0.00	2.07 ± 0.08	1.28 ± 0.01	1.58 ± 0.09	2.1 ± 0.02	2.43 ± 0.00	1.21 ± 0.01	1.83
Glycine	0.69 ± 0.05	0.73 ± 0.00	0.69 ± 0.00	0.85 ± 0.00	0.87 ± 0.03	0.57 ± 0.00	0.70 ± 0.04	1.00 ± 0.01	1.14 ± 0.00	0.59 ± 0.00	0.83
Proline	0.54 ± 0.04	0.66 ± 0.00	0.59 ± 0.01	0.69 ± 0.00	0.74 ± 0.04	0.51 ± 0.01	0.63 ± 0.03	0.83 ± 0.01	0.98 ± 0.00	0.54 ± 0.00	0.71
Serine	0.69 ± 0.05	0.74 ± 0.02	0.66 ± 0.01	0.83 ± 0.00	0.87 ± 0.04	0.56 ± 0.00	0.70 ± 0.04	1.01 ± 0.01	1.23 ± 0.00	0.58 ± 0.01	0.85
Tyrosine	0.31 ± 0.01	0.31 ± 0.00	0.27 ± 0.00	0.34 ± 0.00	0.41 ± 0.00	0.24 ± 0.00	0.24 ± 0.02	0.50 ± 0.01	0.58 ± 0.00	0.25 ± 0.01	0.38
EAA ratio (%)	47.5	47.41	48.98	48.73	48.33	49.31	48.63	49.45	48.8	49.34	48.65
AAS											
Histidine	0.83	0.78	0.84	0.81	0.83	0.79	0.86	0.66	0.77	0.79	0.80
Isoleucine	0.90	0.91	0.93	0.90	0.90	0.92	0.88	0.93	0.92	0.88	0.91
Leucine	1	1	1	1	1	1	1	0.98	0.96	0.98	0.99
Lysine	1	1	1	1	1	1	1	1	1	1	1
Phenylalanine + Tyrosine	0.93	0.86	0.84	0.84	0.88	0.86	0.85	0.90	0.91	0.89	0.88
Threonine	0.93	1	1	1	1	1	1	1	1	1	0.99
Valine	1	0.98	0.98	0.94	0.93	0.96	0.94	0.99	0.97	0.95	0.96
EAAI	0.94	0.93	0.94	0.93	0.93	0.93	0.93	0.92	0.93	0.93	0.93
	**Bangiales**										**Rhodymeniales**
	***Por21***	***Por23***	***Por28***	***Pyr22***	***Pyr26***	***Pyr31***	***Pyr32***	***Pyr33***	***Pyr36***	**Mean**	***Rcor10***
Essential amino acids											
Arginine	1.63 ± 0.06	1.53 ± 0.11	1.89 ± 0.07	1.41 ± 0.00	1.42 ± 0.10	1.18 ± 0.03	1.09 ± 0.11	0.48 ± 0.56	1.36 ± 0.00	1.33	1.66 ± 0.06
Histidine	0.43 ± 0.02	0.38 ± 0.02	0.48 ± 0.01	0.34 ± 0.00	0.38 ± 0.03	0.31 ± 0.01	0.26 ± 0.03	0.12 ± 0.13	0.34 ± 0.00	0.34	0.38 ± 0.00
Isoleucine	1.13 ± 0.04	1.00 ± 0.06	1.26 ± 0.00	0.82 ± 0.00	0.94 ± 0.07	0.81 ± 0.00	0.67 ± 0.08	0.30 ± 0.35	0.84 ± 0.01	0.86	1.14 ± 0.01
Leucine	2.09 ± 0.08	1.87 ± 0.11	2.51 ± 0.02	1.49 ± 0.00	1.89 ± 0.13	1.5 ± 0.01	1.24 ± 0.14	0.56 ± 0.64	1.54 ± 0.01	1.63	1.62 ± 0.04
Lysine	1.45 ± 0.06	1.41 ± 0.09	1.64 ± 0.01	1.38 ± 0.00	1.34 ± 0.10	1.09 ± 0.00	1.06 ± 0.13	0.48 ± 0.55	1.37 ± 0.02	1.25	1.40 ± 0.03
Methionine	0.38 ± 0.00	0.33 ± 0.05	0.57 ± 0.04	0.17 ± 0.00	0.31 ± 0.03	0.2 ± 0.05	0.19 ± 0.02	0.05 ± 0.05	0.15 ± 0.00	0.26	0.34 ± 0.01
Phenylalanine	1.14 ± 0.06	1.09 ± 0.07	1.25 ± 0.02	0.89 ± 0.00	0.96 ± 0.07	0.86 ± 0.00	0.74 ± 0.08	0.34 ± 0.39	0.9 ± 0.01	0.91	1.13 ± 0.04
Threonine	1.56 ± 0.07	1.39 ± 0.08	1.70 ± 0.05	1.24 ± 0.00	1.28 ± 0.08	1.23 ± 0.00	0.98 ± 0.10	0.46 ± 0.53	1.22 ± 0.00	1.23	1.15 ± 0.05
Valine	1.72 ± 0.06	1.64 ± 0.10	1.89 ± 0.03	1.48 ± 0.00	1.52 ± 0.12	1.29 ± 0.01	1.2 ± 0.13	0.57 ± 0.66	1.52 ± 0.01	1.43	1.34 ± 0.01
Non-essential amino acids											
Alanine	3.10 ± 0.12	2.43 ± 0.13	3.75 ± 0.09	2.42 ± 0.01	3.09 ± 0.26	2.02 ± 0.01	1.95 ± 0.20	0.76 ± 0.88	2.37 ± 0.01	2.43	1.69 ± 0.02
Aspartic acid	2.75 ± 0.13	2.43 ± 0.15	3.13 ± 0.03	2.17 ± 0.00	2.38 ± 0.18	2.00 ± 0.03	1.70 ± 0.18	0.78 ± 0.90	2.24 ± 0.00	2.18	2.61 ± 0.08
Glutamic acid	2.83 ± 0.14	2.52 ± 0.16	3.57 ± 0.04	2.21 ± 0.01	2.57 ± 0.19	2.20 ± 0.00	1.78 ± 0.19	0.78 ± 0.90	2.41 ± 0.01	2.32	2.64 ± 0.08
Glycine	1.80 ± 0.08	1.78 ± 0.10	1.96 ± 0.03	1.72 ± 0.00	1.67 ± 0.12	1.55 ± 0.04	1.36 ± 0.17	0.64 ± 0.75	1.71 ± 0.02	1.58	1.23 ± 0.04
Proline	1.21 ± 0.05	1.20 ± 0.07	1.33 ± 0.03	0.98 ± 0.00	1.02 ± 0.07	0.95 ± 0.00	0.78 ± 0.08	0.37 ± 0.43	0.98 ± 0.00	0.98	0.99 ± 0.03
Serine	1.32 ± 0.08	1.20 ± 0.08	1.51 ± 0.03	1.14 ± 0.00	1.23 ± 0.09	0.97 ± 0.00	0.91 ± 0.09	0.41 ± 0.48	1.19 ± 0.00	1.10	1.40 ± 0.05
Tyrosine	0.62 ± 0.05	0.60 ± 0.12	0.87 ± 0.11	0.52 ± 0.00	0.57 ± 0.07	0.45 ± 0.08	0.48 ± 0.01	0.18 ± 0.21	0.44 ± 0.00	0.53	0.70 ± 0.00
EAA ratio (%)	45.83	46.65	45.02	45.22	44.48	45.46	45.32	46.16	44.88	45.45	45.32
AAS											
Histidine	0.81	0.77	0.77	0.78	0.8	0.77	0.75	0.73	0.78	0.77	0.81
Isoleucine	0.76	0.73	0.74	0.69	0.73	0.73	0.69	0.69	0.71	0.72	0.89
Leucine	1	1	1	0.94	1	1	0.96	0.95	0.97	0.98	0.94
Lysine	1	1	1	1	1	1	1	1	1	1	1
Phenylalanine + Tyrosine	0.78	0.82	0.83	0.79	0.78	0.79	0.83	0.78	0.74	0.79	0.93
Threonine	1	1	1	1	1	1	1	1	1	1	1
Valine	1	1	1	1	1	1	1	1	1	1	0.95
EAAI	0.90	0.90	0.90	0.88	0.89	0.89	0.88	0.87	0.88	0.89	0.93

The results are means of triplicate determination ± standard deviation.

**Table 4:** Amino acid concentration (g/100 g), essential amino acid ratio (EAA ratio), amino acid score (AAS), and essential amino acid index (EAAI) of lyophilized species of green macroalgae.

	Bryopsidales			Ulvales						
	*Cod15*	*Cod16*	Mean	*Ulva11*	*Ulva18*	*Ulva19*	*Ulva20*	*Ulva30*	*Ulva17*	Mean
Essential amino acids										
Arginine	0.95 ± 0.03	0.66 ± 0.02	0.81	1.35 ± 0.03	1.41 ± 0.01	1.69 ± 0.02	2.07 ± 0.04	1.73 ± 0.00	0.67 ± 0.00	1.49
Histidine	0.30 ± 0.00	0.19 ± 0.00	0.25	0.45 ± 0.02	0.37 ± 0.00	0.40 ± 0.01	0.44 ± 0.01	0.46 ± 0.01	0.15 ± 0.00	0.38
Isoleucine	0.92 ± 0.02	0.70 ± 0.02	0.81	0.94 ± 0.02	1.07 ± 0.00	1.02 ± 0.02	1.18 ± 0.02	1.16 ± 0.03	0.57 ± 0.01	0.99
Leucine	1.51 ± 0.04	1.06 ± 0.04	1.29	1.53 ± 0.04	1.82 ± 0.01	1.70 ± 0.02	1.96 ± 0.04	1.86 ± 0.05	0.92 ± 0.02	1.64
Lysine	1.14 ± 0.00	0.89 ± 0.01	1.02	1.11 ± 0.03	1.08 ± 0.03	1.12 ± 0.01	1.36 ± 0.00	1.20 ± 0.04	0.55 ± 0.02	1.07
Methionine	0.35 ± 0.01	0.24 ± 0.00	0.30	0.36 ± 0.01	0.47 ± 0.00	0.36 ± 0.01	0.46 ± 0.02	0.49 ± 0.00	0.21 ± 0.00	0.39
Phenylalanine	0.96 ± 0.03	0.71 ± 0.03	0.84	1.12 ± 0.03	1.28 ± 0.01	1.29 ± 0.02	1.43 ± 0.02	1.27 ± 0.03	0.68 ± 0.01	1.18
Threonine	1.10 ± 0.00	0.82 ± 0.00	0.96	1.17 ± 0.04	1.17 ± 0.00	1.25 ± 0.06	1.42 ± 0.00	1.44 ± 0.04	0.61 ± 0.01	1.18
Valine	1.29 ± 0.00	1.00 ± 0.02	1.15	1.35 ± 0.02	1.42 ± 0.00	1.47 ± 0.02	1.65 ± 0.03	1.66 ± 0.05	0.76 ± 0.02	1.39
Non-essential amino acids										
Alanine	1.45 ± 0.01	1.14 ± 0.02	1.30	1.96 ± 0.05	1.86 ± 0.03	2.14 ± 0.05	2.38 ± 0.01	2.20 ± 0.06	1.05 ± 0.02	1.93
Aspartic acid	2.20 ± 0.03	1.70 ± 0.00	1.95	2.55 ± 0.09	2.63 ± 0.01	2.75 ± 0.04	3.07 ± 0.01	2.96 ± 0.06	1.38 ± 0.03	2.56
Glutamic acid	2.33 ± 0.05	1.83 ± 0.00	2.08	2.74 ± 0.08	2.61 ± 0.01	3.02 ± 0.02	3.77 ± 0.03	3.38 ± 0.11	1.47 ± 0.04	2.83
Glycine	1.14 ± 0.02	0.88 ± 0.01	1.02	1.47 ± 0.03	1.53 ± 0.00	1.54 ± 0.03	1.76 ± 0.01	1.70 ± 0.08	0.83 ± 0.02	1.47
Proline	0.89 ± 0.02	0.67 ± 0.03	0.78	1.04 ± 0.02	1.08 ± 0.03	1.14 ± 0.02	1.35 ± 0.02	1.13 ± 0.03	0.55 ± 0.01	1.05
Serine	1.10 ± 0.01	0.84 ± 0.00	0.98	1.21 ± 0.06	1.28 ± 0.04	1.33 ± 0.07	1.53 ± 0.02	1.49 ± 0.03	0.66 ± 0.02	1.25
Tyrosine	0.65 ± 0.00	0.49 ± 0.03	0.57	0.72 ± 0.02	0.75 ± 0.00	0.64 ± 0.01	0.68 ± 0.02	0.86 ± 0.00	0.30 ± 0.00	0.66
EAA ratio (%)	46.58	45.38	45.98	44.54	46.22	45.06	45.14	45.12	45.12	45.20
AAS										
Histidine	0.71	0.60	0.65	1	0.78	0.86	0.81	0.88	0.61	0.82
Isoleucine	0.8	0.82	0.81	0.77	0.82	0.79	0.80	0.80	0.86	0.81
Leucine	0.99	0.93	0.96	0.94	1	0.99	1	0.97	1	0.98
Lysine	1	1	1	1	1	1	1	1	1	1
Phenylalanine + Tyrosine	0.93	0.93	0.93	0.99	1	0.98	0.95	0.97	0.98	0.98
Threonine	1	1	1	1	1	1	1	1	1	1
Valine	1	1	1	1	1	1	1	1	1	1
EAAI	0.91	0.88	0.90	0.95	0.94	0.94	0.93	0.94	0.91	0.94

The results are means of triplicate determination ± standard deviation.

**Table 5:** Fatty acid composition of lyophilized species of brown macroalgae from the Peruvian coast. Results expressed in relative percentage.

FA (%)	*Laminariales*										
	*Ecok1 ^F^*	*Egra3 ^F^*	*Ecok4 ^F^*	*Ecok6 ^F^*	*Egra7 ^F^*	Median	*Egra2 ^S^*	*Ecok5 ^S^*	*Egra8 ^S^*	Median	*Lber37 ^F^*
C 14:0	4.94 ± 0.01	8.02 ± 0.31	6.78 ± 0.13	7.37 ± 1.04	6.61 ± 0.52		10.49 ± 0.45	6.31 ± 0.39	10.78 ± 1.53		10.47 ± 0.38
C 16:0	16.41 ± 0.36	14.34 ± 0.51	18.13 ± 0.02	24.78 ± 3.33	17.68 ± 1.17		19.30 ± 0.34	25.06 ± 0.72	20.65 ± 1.36		20.86 ± 0.52
C 18:0	0.71 ± 0.19	1.27 ± 0.17	0.69 ± 0.01	2.49 ± 1.15	1.00 ± 0.35		1.28 ± 0.09	1.51 ± 0.07	1.77 ± 0.69		0.91 ± 0.08
Ʃ SFAs	22.09	23.43	25.60	34.61	25.51	25.51	30.86	32.82	33.33	32.82	32.30
C 16:1n − 9	2.66 ± 0.03	8.98 ± 1.00	5.52 ± 0.02	3.85 ± 0.95	4.90 ± 0.39		1.88 ± 0.03	1.46 ± 0.19	2.34 ± 0.05		5.06 ± 0.01
C 16:1n − 7	7.02 ± 0.22	5.42 ± 0.19	10.07 ± 0.34	11.31 ± 0.16	3.73 ± 0.47		5.63 ± 0.25	7.04 ± 0.10	3.31 ± 0.90		2.06 ± 0.03
C 18:1n − 7	0.39 ± 0.06	0.70 ± 0.01	0.62 ± 0.02	1.66 ± 0.42	0.64 ± 0.15		0.42 ± 0.04	0.49 ± 0.14	0.31 ± 0.13		12.81 ± 0.63
C 18:1n − 9	11.05 ± 0.82	12.52 ± 0.87	13.34 ± 0.26	14.28 ± 0.27	11.75 ± 0.63		23.25 ± 0.34	26.33 ± 0.71	23.73 ± 1.64		0.55 ± 0.38
Ʃ MUFAs	21.18	27.39	29.51	31.25	21.10	27.39	31.27	35.26	29.69	31.27 ^†^	20.35
C 16:4n − 3	0.39 ± 0.10	1.2 ± 0.14	0.47 ± 0.01	1.83 ± 0.53	1.04 ± 0.2		0.24 ± 0.01	ND	0.29 ± 0.04		0.82 ± 0.49
C 18:2n − 6	6.41 ± 0.12	9.13 ± 0.13	6.01 ± 0.06	4.57 ± 0.32	6.85 ± 0.24		7.70 ± 0.28	3.74 ± 0.02	4.74 ± 0.29		4.07 ± 0.00
C 18:3n − 6	2.94 ± 0.10	1.58 ± 0.08	3.19 ± 0.13	1.54 ± 0.12	1.79 ± 0.10		2.97 ± 0.03	1.46 ± 0.08	1.88 ± 0.12		0.38 ± 0.02
C 18:3n − 3	5.52 ± 0.55	7.54 ± 0.49	3.27 ± 0.10	4.14 ± 1.27	8.02 ± 0.57	5.50	1.81 ± 0.22	0.87 ± 0.02	0.93 ± 0.07	0.96	7.41 ± 0.70
C 18:4n − 3	14.28 ± 0.87	8.89 ± 0.41	8.74 ± 0.45	4.79 ± 0.9	11.38 ± 0.71	8.91	2.90 ± 0.67	1.51 ± 0.36	1.44 ± 0.13	1.53	9.5 ± 0.77
C 20:4n − 3	0.32 ± 0.00	0.89 ± 0.04	0.38 ± 0.02	0.30 ± 0.01	0.78 ± 0.05		0.28 ± 0.00	0.16 ± 0.01	0.21 ± 0.00		1.09 ± 0.00
C 20:4n − 6	17.85 ± 0.36	14.23 ± 0.48	15.24 ± 0.24	12.03 ± 1.73	17.4 ± 0.65	15.21	18.45 ± 0.1	20.1 ± 1.09	24.53 ± 2.52	20.06	13.91 ± 0.44
C 20:5n − 3	8.74 ± 0.29	5.3 ± 0.3	7.56 ± 0.22	5.05 ± 0.53	6.43 ± 0.37	6.46	3.39 ± 0.31	3.96 ± 0.06	3.08 ± 0.23	3.40	10.09 ± 0.28
C 22:6n − 3	ND	ND	ND	ND	ND		ND	ND	ND		ND
Ʃ PUFAs	56.37	49.17	44.86	34.14	53.38	48.25	37.86	31.91	36.98	36.98	47.33
ɷ3	29.15	24.09	20.47	16.11	27.56	24.09	8.71	6.68	5.94	6.68	29.19
ɷ6	27.22	25.08	24.39	18.03	25.82	25.08	29.15	25.23	31.04	29.15	18.14
ɷ6/ɷ3	0.93	1.04	1.19	1.12	0.94	0.99	3.35	3.78	5.23	3.78 ^††^	0.62

The results are means of triplicate determination ± standard deviation. Medians were compared by Kruskal–Wallis test. *^F^* Frond; *^S^* stipe; ^†^ *z*-value = 2.50 (*p* = 0.006); ^††^ *z*-value = 2.56 (*p* = 0.001). ND: Not detected.

**Table 6:** Fatty acid composition of lyophilized species of red macroalgae from the Peruvian coast. Results expressed in relative percentage.

FA (%)	*Gigartinales*											
	*Ccha12*	*Ccha24*	*Ccha27*	*Ccha29*	*Ccha34*	*Ccha9*	*Ccha25*	Median	*Cvar14*	*Cvar35*	Median	*Mcan13*
C 14:0	ND	3.21 ± 0.86	2.52 ± 0.38	3.40 ± 0.64	3.52 ± 0.24	3.74 ± 0.12	3.21 ± 0.16		ND	1.92 ± 0.09		ND
C 16:0	42.24 ± 1.11	39.20 ± 0.82	35.42 ± 0.33	42.13 ± 0.89	43.27 ± 1.06	31.15 ± 0.02	42.15 ± 0.86		20.9 ± 0.08	15.89 ± 0.32		41.83 ± 1.17
C 18:0	4.67 ± 0.16	1.54 ± 0.01	1.15 ± 0.07	1.31 ± 0.08	2.04 ± 0.22	2.05 ± 0.58	1.75 ± 0.11		0.69 ± 0.01	0.6 ± 0.22		5.46 ± 0.31
Ʃ SFAs	47.62	44.33	39.61	46.98	49.2	36.79	47.62	46.98 ^‡^	21.61	18.46	20.04	45.00
C 16:1n − 9	5.67 ± 1.30	2.56 ± 0.13	2.50 ± 0.09	3.14 ± 0.09	2.30 ± 0.08	2.75 ± 0.16	2.78 ± 0.01		3.61 ± 0.13	3.23 ± 0.05		4.73 ± 0.15
C 16:1n − 7	2.29 ± 0.31	1.23 ± 0.01	1.66 ± 0.09	1.79 ± 0.03	1.24 ± 0.03	1.58 ± 0.31	1.56 ± 0.12		2.86 ± 0.25	1.81 ± 0.02		3.35 ± 0.33
C 18:1n − 7	4.06 ± 1.26	1.12 ± 0.09	1.17 ± 0.07	1.12 ± 0.03	1.47 ± 0.20	11.61 ± 0.14	1.16 ± 0.01		ND	1.05 ± 0.05		ND
C 18:1n − 9	20.62 ± 1.20	13.29 ± 0.06	11.49 ± 0.06	13.64 ± 0.06	17.97 ± 0.26	1.10 ± 0.25	14.17 ± 0.10		4.75 ± 0.12	4.22 ± 0.06		19.64 ± 0.91
Ʃ MUFAs	28.57	18.04	17.54	19.88	23.38	16.98	19.73	19.73	11.27	10.27	10.77	30.00
C 16:4n − 3	ND	0.23 ± 0.02	0.18 ± 0.00	0.26 ± 0.01	0.17 ± 0.01	ND	0.27 ± 0.02		0.35 ± 0.04	0.28 ± 0.02		ND
C 18:2n − 6	3.55 ± 0.46	1.54 ± 0.12	1.59 ± 0.05	1.19 ± 0.04	2.01 ± 0.06	1.47 ± 0.16	1.31 ± 0.04		0.72 ± 0.03	0.62 ± 0.01		2.28 ± 0.13
C 18:3n − 6	ND	0.39 ± 0.01	0.62 ± 0.07	0.59 ± 0.00	0.73 ± 0.05	0.51 ± 0.05	0.31 ± 0.02		1.89 ± 0.03	4.96 ± 0.14		ND
C 18:3n − 3	2.98 ± 2.54	0.15 ± 0.05	0.14 ± 0.03	0.12 ± 0.02	ND	ND	ND	0.15	0.95 ± 0.17	0.39 ± 0.00	0.62	ND
C 18:4n − 3	ND	0.15 ± 0.02	0.16 ± 0.01	0.35 ± 0.08	ND	ND	0.32 ± 0.07	0.24	0.79 ± 0.12	0.21 ± 0.00	0.42	ND
C 20:4n − 3	ND	0.16 ± 0.02	ND	ND	ND	ND	ND		0.12 ± 0.00	0.06 ± 0.01		ND
C 20:4n − 6	5.36 ± 1.47	9.37 ± 0.62	8.06 ± 0.15	8.59 ± 0.13	8.13 ± 0.20	12.25 ± 0.65	10.86 ± 0.33	8.43	51.11 ± 0.46	58.3 ± 0.53	54.73 ^‡‡‡^	8.69 ± 0.73
C 20:5n − 3	8.55 ± 1.63	25.87 ± 0.82	33.36 ± 0.17	22.37 ± 0.30	17.17 ± 0.74	31.78 ± 0.13	20.15 ± 1.10	22.29	9.91 ± 0.32	6.46 ± 0.12	8.16	10.77 ± 0.03
C 22:6n − 3	ND	ND	ND	ND	ND	ND	ND		0.19 ± 0.04	ND		ND
Ʃ PUFAs	23.8	37.64	42.86	33.12	27.42	46.23	32.65	33.12	65.98	71.27	68.23	20.00
ɷ3	14.28	26.29	33.12	22.89	16.94	32.08	20.41	22.89	12.42	7.22	9.82	10.00
ɷ6	9.52	11.35	9.74	10.23	10.48	14.15	12.24	10.48	53.56	64.05	58.81	10.00
ɷ6/ɷ3	0.67	0.43	0.29	0.45	0.62	0.44	0.60	0.45	4.31	8.87	6.59 ^‡‡^	1.00
The results are means of triplicate determination ± standard deviation. Medians were compared by Kruskal–Wallis test. ^‡^ *z*-value = 3.41 (*p* = 0.008); ^‡‡^ *z*-value = 2.28 (*p* = 0.001); ^‡‡‡^ *z*-value = 2.35 (*p* = 0.001). ND: Not detected.												
**FA (%)**	***Bangiales***										**Rhodymeniales**	
	***Por21***	***Por23***	***Por28***	***Pyr22***	***Pyr26***	***Pyr31***	***Pyr32***	***Pyr33***	***Pyr36***	**Median**	**Rcor10**	
C 14:0	0.38 ± 0.02	0.71 ± 0.08	0.28 ± 0.02	0.82 ± 0.02	0.38 ± 0.03	0.69 ± 0.04	1.71 ± 0.11	3.24 ± 0.01	1.33 ± 0.13		6.50 ± 0.15	
C 16:0	33.89 ± 0.18	35.96 ± 0.05	31.98 ± 0.63	33.87 ± 0.04	30.36 ± 1.28	32.87 ± 0.10	36.63 ± 0.24	37.27 ± 0.80	29.85 ± 0.4		31.08 ± 0.04	
C 18:0	1.10 ± 0.03	1.29 ± 0.03	1.12 ± 0.01	1.27 ± 0.05	1.02 ± 0.03	1.41 ± 0.02	1.43 ± 0.04	3.72 ± 2.71	1.29 ± 0.09		1.25 ± 0.26	
Ʃ SFAs	35.46	37.90	33.59	35.88	31.62	34.71	40.00	44.94	32.48	31.46	38.96	
C 16:1n − 9	3.75 ± 0.05	4.42 ± 0.01	3.47 ± 0.07	3.23 ± 0.17	2.99 ± 0.01	2.89 ± 0.01	2.71 ± 0.06	1.85 ± 0.13	2.45 ± 0.02		5.03 ± 0.22	
C 16:1n − 7	0.59 ± 0.02	0.97 ± 0.09	0.54 ± 0.01	2.27 ± 0.10	0.89 ± 0.01	2.92 ± 0.18	8.19 ± 1.55	18.82 ± 0.36	4.46 ± 0.24		6.01 ± 0.02	
C 18:1n − 7	3.13 ± 0.11	3.29 ± 0.07	3.4 ± 0.03	2.36 ± 0.03	3.15 ± 0.05	4.00 ± 0.06	3.37 ± 0.20	3.01 ± 0.35	3.42 ± 0.05		2.03 ± 0.01	
C 18:1n − 9	1.48 ± 0.21	2.29 ± 0.05	1.25 ± 0.02	3.54 ± 0.09	1.75 ± 0.04	2.60 ± 0.22	2.93 ± 0.12	4.00 ± 0.16	3.06 ± 0.02		8.45 ± 0.47	
Ʃ MUFAs	9.01	10.83	8.58	11.4	8.54	12.39	17.4	27.53	13.92	11.40	21.52	
C 16:4n − 3	0.35 ± 0.04	0.36 ± 0.05	0.31 ± 0.01	0.28 ± 0.04	0.24 ± 0.01	0.26 ± 0.01	0.31 ± 0.06	0.36 ± 0.00	0.23 ± 0.01		0.36 ± 0.08	
C 18:2n − 6	2.93 ± 0.10	2.81 ± 0.01	2.52 ± 0.03	2.41 ± 0.00	2.12 ± 0.12	2.77 ± 0.07	2.54 ± 0.08	2.16 ± 0.02	3.56 ± 0.42		1.14 ± 0.09	
C 18:3n − 6	0.30 ± 0.01	0.31 ± 0.01	0.68 ± 0.02	0.42 ± 0.00	0.73 ± 0.00	0.38 ± 0.02	0.54 ± 0.04	0.46 ± 0.01	0.52 ± 0.02		0.62 ± 0.03	
C 18:3n − 3	0.30 ± 0.17	0.18 ± 0.02	ND	0.23 ± 0.01	ND	0.34 ± 0.02	0.47 ± 0.06	0.21 ± 0.04	0.36 ± 0.00	0.41 ^‡‡‡^	0.15 ± 0.00	
C 18:4n − 3	0.15 ± 0.14	0.05 ± 0.00	0.08 ± 0.01	ND	0.15 ± 0.00	ND	0.43 ± 0.06	0.21 ± 0.00	0.09 ± 0.02		0.25 ± 0.00	
C 20:4n − 3	0.29 ± 0.01	0.25 ± 0.01	0.29 ± 0.00	0.41 ± 0.02	1.36 ± 0.02	0.09 ± 0.00	0.35 ± 0.00	0.19 ± 0.01	0.45 ± 0.06		ND	
C 20:4n − 6	24.03 ± 0.52	25.13 ± 0.20	21.13 ± 0.44	14.07 ± 0.14	8.39 ± 0.30	29.46 ± 0.56	12.11 ± 0.11	10.26 ± 0.52	14.17 ± 0.39	14.13	9.85 ± 0.24	
C 20:5n − 3	27.31 ± 0.58	21.99 ± 0.04	32.95 ± 0.28	34.83 ± 0.10	46.46 ± 1.16	19.32 ± 0.08	26.27 ± 1.61	14.24 ± 1.11	34.75 ± 1.65	27.33 ^‡^	26.42 ± 0.54	
C 22:6n − 3	ND	ND	ND	ND	ND	ND	ND	ND	ND		ND	
Ʃ PUFAs	55.53	51.28	57.86	52.70	59.83	52.88	42.61	27.52 *	53.63	52.88 ^‡‡^	38.78	
ɷ3	28.2	22.93	33.59	35.86	48.29	20.24	26.96	14.6	35.58	28.20	27.28	
ɷ6	27.33	28.35	24.27	16.84	11.54	32.64	15.65	12.92	18.05	18.05	11.5	
ɷ6/ɷ3	0.97	1.24	0.72	0.47	0.24	1.61	0.58	0.88	0.51	0.72	0.42	

The results are means of triplicate determination ± standard deviation. Medians were compared by Kruskal–Wallis test. * Outlier; ^‡^ *z*-value = 3.58 (*p* = 0.000); ^‡‡^ *z*-value = 2.32 (*p* = 0.031); ^‡‡‡^ *z*-value = −2.86 (*p* = 0.004). ND: Not detected.

**Table 7:** Fatty acid composition of lyophilized species of green macroalgae from the Peruvian coast. Results expressed in relative percentage.

FA (%)	*Bryopsidales*			*Ulvales*						
	*Cod15*	*Cod16*	Median	*Ulva11*	*Ulva18*	*Ulva19*	*Ulva20*	*Ulva30*	*Ulva17*	Median
C 14:0	ND	ND		0.37 ± 0.05	ND	0.79 ± 0.03	0.63 ± 0.02	0.84 ± 0.01	ND	
C 16:0	29.8 ± 0.29	31.57 ± 0.69		15.48 ± 0.09	24.87 ± 0.5	40.71 ± 0.20	39.6 ± 0.75	21.53 ± 0.10	44.11 ± 0.03	
C 18:0	0.89 ± 0.02	1.03 ± 0.01		0.29 ± 0.11	0.37 ± 0.00	0.84 ± 0.01	0.86 ± 0.06	0.27 ± 0.01	0.88 ± 0.02	
Ʃ SFAs	30.70	32.50	31.60	16.13	24.79	42.23	41.23	22.69	44.98	33.01
C 16:1n − 9	7.83 ± 0.41	9.19 ± 0.43		5.8 ± 0.23	8.37 ± 0.63	11.12 ± 0.2	7.36 ± 0.10	6.66 ± 0.52	14.36 ± 0.22	
C 16:1n − 7	1.11 ± 0.03	1.02 ± 1.18		0.82 ± 0.06	0.78 ± 0.01	2.54 ± 0.17	2.13 ± 0.04	2.18 ± 0.40	1.68 ± 0.09	
C 18:1n − 7	0.56 ± 0.05	ND		1.10 ± 0.10	9.40 ± 0.18	16.83 ± 0.07	14.87 ± 0.11	12.63 ± 0.02	17.56 ± 0.43	
C 18:1n − 9	16.65 ± 0.26	15.21 ± 0.09		6.16 ± 0.02	0.75 ± 0.04	1.46 ± 0.04	1.48 ± 0.02	1.04 ± 0.06	1.66 ± 0.52	
Ʃ MUFAs	26.16	25.44	25.80	13.90	19.01	32.06	25.73	22.44	34.95	24.09
C 16:4n − 3	0.37 ± 0.02	0.49 ± 0.03		16.35 ± 0.18	15.65 ± 0.19	2.69 ± 0.01	3.14 ± 0.05	15.1 ± 0.34	3.92 ± 0.44	
C 18:2n − 6	6.84 ± 0.02	8.75 ± 0.06		5.42 ± 0.09	3.37 ± 0.29	5.65 ± 0.09	6.83 ± 0.08	5.11 ± 0.00	2.71 ± 0.22	
C 18:3n − 6	2.15 ± 0.02	2.06 ± 0.03		0.86 ± 0.05	0.60 ± 0.05	0.73 ± 0.01	1.10 ± 0.03	0.67 ± 0.02	0.31 ± 0.06	
C 18:3n − 3	22.88 ± 0.89	18.14 ± 0.08	20.54 ^‡‡‡^	26.17 ± 0.12	13.99 ± 0.54	8.72 ± 0.06	11.13 ± 0.11	20.42 ± 0.18	6.57 ± 0.31	12.58 ^‡‡‡‡^
C 18:4n − 3	0.97 ± 0.00	0.98 ± 0.03	0.95	16.76 ± 0.37	17.9 ± 0.10	6.48 ± 0.10	9.20 ± 0.14	11.15 ± 0.24	4.15 ± 0.4	10.08 ^‡‡‡‡‡^
C 20:4n − 3	5.83 ± 0.60	7.36 ± 0.13		0.40 ± 0.00	0.32 ± 0.03	0.72 ± 0.07	0.74 ± 0.03	0.43 ± 0.00	0.40 ± 0.33	
C 20:4n − 6	0.30 ± 0.01	0.24 ± 0.00	6.56	0.51 ± 0.06	0.49 ± 0.03	0.28 ± 0.02	0.41 ± 0.03	1.25 ± 0.81	0.12 ± 0.01	0.37 ^‡^
C 20:5n − 3	3.67 ± 0.08	3.74 ± 0.07	3.72	0.79 ± 0.15	0.54 ± 0.02	0.45 ± 0.03	0.52 ± 0.08	0.72 ± 0.09	0.70 ± 0.08	0.68 ^‡‡^
C 22:5n − 3	0.05 ± 0.00	0.05 ± 0.01		2.72 ± 0.08	2.60 ± 0.23	ND	ND	ND	0.75 ± 0.11	
C 22:6n − 3	ND	ND		ND	ND	ND	ND	ND	ND	
Ʃ PUFAs	43.05	41.87	42.46	69.98	56.21	25.69	33.04	54.86	19.88	43.95
ɷ3	28.27	23.69	25.98	63.32	52.07	19.08	24.85	47.8	16.27	36.33
ɷ6	14.78	18.18	16.48	6.66	4.14	6.61	8.19	7.06	3.61	6.64
ɷ6/ɷ3	0.52	0.77	0.65	0.11	0.08	0.35	0.33	0.15	0.22	0.19

The results are means of triplicate determination ± standard deviation. Medians were compared by Kruskal–Wallis test. ^‡^ *z*-value = −3.55 (*p* = 0.001); ^‡‡^ *z*-value = −3.83 (*p* = 0.000); ^‡‡‡^ *z*-value = 2.02 (*p* = 0.004); ^‡‡‡‡^ *z*-value = 2.92 (*p* = 0.004); ^‡‡‡‡‡^ *z*-value = 2.26 (*p* = 0.024). ND: Not detected.

**Table 8:** Taxonomic position and collection data of Peruvian seaweed included in this study.

	Order	Species	Code	Locality	GPS Coordinates
Brown Algae	Laminariales	*Eisenia cokeri* M.Howe	*Ecok1 ^F^*	Cabeza de Leon, Isla Lobos de Tierra	06°25′31.4′′, 80°51′59.2′′
		*Eisenia cokeri*	*Ecok4 ^F^*	La Gramita, Casma	09°42′24.2′′, 78°17′48.4′′
		*Eisenia cokeri*	*Ecok5 ^S^*	El Ancla, Laguna Grande, Paracas	14°10′29.5′′, 76°15′24.8′′
		*Eisenia cokeri*	*Ecok6 ^F^*	El Ancla, Laguna Grande, Paracas	14°10′29.5′′, 76°15′24.8′′
		*Eisenia gracilis* E.Y.Dawson, Acleto & Foldvik	*Egra2 ^S^*	Playa Tres Hermanas, Marcona	15°26′29.1′′, 75°04′32.5′′
		*Eisenia gracilis*	*Egra3 ^F^*	Playa Tres Hermanas, Marcona	15°26′29.1′′, 75°04′32.5′′
		*Eisenia gracilis*	*Egra7 ^F^*	Leonas, Ilo	17°40′39.3”, 71°22′15.9”
		*Eisenia gracilis*	*Egra8 ^S^*	Leonas, Ilo	17°40′39.3”, 71°22′15.9”
		*Lessonia berteroana* Montagne	*Lber37 ^F^*	Playa Punta San Juanito, Marcona	15°16′18.9”, 75°14′26.9”
Red Algae	Gigartinales	*Chondracanthus chamissoi* (C. Agardh) Kützing	*Ccha12*	Playa Mendieta, Paracas	14°02′48”, 76°15′51.2”
		*Chondracanthus chamissoi*	*Ccha24*	Playa Guaynuná, Casma	09°21′01”, 78°25′33.1”
		*Chondracanthus chamissoi*	*Ccha27*	Playa San Francisco Grande, Ancón	11°46′12.45”, 77°11′27.08”
		*Chondracanthus chamissoi*	*Ccha29*	Playa Las Ninfas, Pucusana	12°28′, 76°48′
		*Chondracanthus chamissoi*	*Ccha34*	Playa Siete Huecos, Marcona	15°24′21.2”, 75°07′54”
		*Chondracanthus chamissoi*	*Ccha9*	Playa Cantolao, Callao	14°02′48”, 76°15′51.2”
		*Chondracanthus chamissoi*	*Ccha25*	Playa La Mesa, Casma	09°46′33.8”, 78°14′42.5”
		*Callophyllis variegata* (Bory) Kützing	*Cvar14*	Playa Mendieta, Paracas	14°02′48”, 76°15′51.2”
		*Callophyllis variegata*	*Cvar35*	Playa Hermosa, Marcona	15°21′13.9”, 75°10′3.7”
		*Mazzaella canaliculata* (C.Agardh) Arakaki & M.E.Ramírez	*Mcan13*	Playa Mendieta, Paracas	14°02′48”, 76°15′51.2”
	Bangiales	*Porphyra* sp.	*Por21*	Playa La Mesa, Casma	09°46′33.8”, 78°14′42.5”
		*Porphyra* sp.	*Por23*	Playa La Mesa, Casma	09°46′36.8”, 78°14′32.4”
		*Porphyra* sp.	*Por28*	Playa Las Ninfas, Pucusana	12°28′, 76°48′
		*Pyropia* sp.	*Pyr22*	Playa La Mesa, Casma	09°46′33.8”, 78°14′42.5”
		*Pyropia* sp.	*Pyr26*	San Francisco Chico, Ancón	11°46′15.65”, 77°11′19.64”
		*Pyropia* sp.	*Pyr31*	Playa Los Leones, Marcona	15°23′09” S, 75°09′30.1”
		*Pyropia* sp.	*Pyr32*	Playa Trompa de Elefante, Marcona	15°23′34.3”, 75°09′32.1”
		*Pyropia* sp.	*Pyr33*	Playa Lobo Fino, Marcona	15°24′25.3”, 75°08′24”
		*Pyropia* sp.	*Pyr36*	Playa Punta San Juanito, Marcona	15°16′18.9”, 75°14′26.9”
	Rhodymeniales	*Rhodymenia corallina* (Bory) Greville	*Rcor10*	Playa Cantolao, Callao	14°02′48”, 76°15′51.2”
Green Algae	Bryopsidales	*Codium* sp.	*Cod15*	Playa Mendieta, Paracas	14°02′48”, 76°15′51.2”
		*Codium* sp.	*Cod16*	Atenas, Bahía de Paracas	13°49′52.1′′, 78°17′52.4′′
	Ulvales	*Ulva* sp.	*Ulva11*	Playa Cantolao, Callao	14°02′48”, 76°15′51.2”
		*Ulva* sp.	*Ulva18*	Playa Mendieta, Paracas	14°02′48”, 76°15′51.2”
		*Ulva* sp.	*Ulva19*	Playa Guaynuná, Casma	09°21′01”, 78°25′33.1”
		*Ulva* sp.	*Ulva20*	Playa Guaynuná, Casma	09°21′01”, 78°25′33.1”
		*Ulva* sp.	*Ulva30*	Playa Las Ninfas, Pucusana	12°28′, 76°48′
		*Ulva papenfussii* Pham-Hoang Hô	*Ulva17*	Atenas, Bahía de Paracas	13°49′52.1′′, 78°17′52.4′′

*^F^* Frond; *^S^* stipe.

## Data Availability

Not applicable.
